# Lab-scale and on-field industrial composting of biodegradable plastic blends for packaging

**DOI:** 10.12688/openreseurope.14893.1

**Published:** 2022-08-23

**Authors:** Zhi Kai Chong, Alexander Hofmann, Marie Haye, Sharon Wilson, Ihsanullah Sohoo, Kerstin Kuchta

**Affiliations:** 1 Circular Resource Engineering and Management (CREM), Hamburg University of Technology, Hamburg, 21073, Germany; 2 Department of Energy and Environmental Engineering (GEn), Institut National des Sciences Appliquées de Lyon, Villeurbanne, 69100, France

**Keywords:** Biodegradable plastic, compostable plastic, compostable packaging, industrial composting, polylactic acid, polybutylene succinate

## Abstract

**Background:** The acceptance of compostable plastic packaging in industrial composting plants is not universal despite available certification for compostability due to the persistence of compostable plastic residues reported by some industrial plants. This study aims to better understand this discrepancy by comparing the disintegration rate of two compostable plastic blends designed for rigid packaging (polylactic acid based) and soft packaging (polybutylene succinate based) between a controlled lab-scale test and an on-field test in an industrial composting plant.

**Methods:** The thermophilic lab-scale disintegration test was conducted according to ISO 20200 in triplicates for 4, 8 and 12 weeks while the on-field test was conducted by exposing duplicate test material in the compost pile of an industrial composting plant in northern Germany, for three weeks. The mass change of the remaining test material >2mm was used as an indicator of disintegration.

**Results:** The rigid packaging blend (1 mm thickness) retained on average 76.4%, 59.0% and 55.7% of its mass after 4, 8 and 12 weeks respectively in the lab-scale test. After exposure to industrial composting on-field, the remaining mass was 97.2% and 99.5%. The soft packaging blend (109±9 µm sample thickness) retained on average 45.4%, 10.9% and 0.3% of its mass after 4, 8 and 12 weeks respectively and 94.0% and 93.8% after exposure to industrial composting on-field.

**Conclusions:** The results show a substantial difference in disintegration rates between the lab-scale and the on-field test after three to four weeks. The difference between the tests that might contribute significantly to the differing disintegration rates is the composition of the composting substrate. Besides the design and characteristics of the packaging itself, the composting substrate and thermophilic composting duration of individual plants are important to determine the suitability of treating compostable plastic packaging in industrial composting plants as well as inform potential solutions.

## Plain language summary

There is resistance from industrial composting plants to the treatment of compostable biodegradable plastics. This study aims to compare the disintegration rates of two new biodegradable plastic blends developed for rigid and soft packaging applications in controlled industrial composting conditions in the lab as well as actual conditions in an industrial composting plant. Results show stark differences between the disintegration of the samples based on mass loss in the lab and in the plant. The 1 mm thick polylactic acid-based blend for rigid packaging experienced much higher disintegration in the lab with 76.4% remaining mass after 4 weeks compared to 97.2% remaining mass after 3 weeks in the industrial composting plant. The 109 µm thick polybutylene succinate-based blend for soft packaging also experienced higher degradation in the lab with 45.4% remaining mass after 4 weeks in the lab compared to 93.8% remaining mass in the industrial composting plant. The shorter high-temperature composting duration in industrial plants as well as the characteristics of their biowaste inputs are potential causes of the large difference. The study highlights the need to better understand the real-world industrial composting conditions and their variations when evaluating composting as a treatment method for biodegradable plastics.

## Introduction

The prevalence of plastic waste and micro-plastic in the environment has spurred research and development of alternatives such as biodegradable plastics. Biodegradable plastics include polymers that can be broken down and mineralized by microbial action. They can be produced from both fossil-based and bio-based resources. It is important to note that compostable plastics are a subcategory of biodegradable plastics that would only disintegrate significantly in specific composting conditions, in contrast to general environmental conditions on land or sea. Thus, they need to be kept in controlled closed systems. Commonly available bio-based biodegradable plastics include polylactic acid (PLA) and polybutylene succinate (PBS) ( IfBB, 2020).

Industrial compostable products are designed to disintegrate within a reasonable period under industrial composting conditions. The major characteristic of industrial composting is the ability to achieve thermophilic temperatures ( Barrena *et al*., 2014; Sundberg *et al*., 2004), in the range from 55°C to 75°C. The composting route might be an alternative for the treatment of plastic waste, for example when mechanical recycling is difficult, such as in the case of multilayer packaging ( de Mello Soares *et al*., 2022; Trinh *et al*., 2021). However, industrial composting primarily aims to treat and stabilize biowaste and produce compost, an agricultural substrate. Thus, the feasibility of the treatment of biodegradable plastics within these plants is not a given.

In Germany, there is no widespread acceptance and treatment of compostable plastics in industrial composting plants ( ANS e.V. (ANS) *et al*., 2019). One of the major issues quoted is the insufficient decomposition of biodegradable plastic products within the normal operating conditions of the plants. This occurs even with products certified industrially compostable via international standards such as EN 13432. The reason is the wide range of different conditions in which industrial composting operators run their plants, which are sometimes very different from the compostable certification conditions ( Di Bartolo *et al*., 2021; Hann *et al*., 2020). The disintegration condition in EN 13432 requires that the product disintegrates under industrial composting conditions and leaves behind less than 10% of the material (> 2 mm) by mass after a maximum of 12 weeks. The industrial composting plants in Germany on the other hand, have an active composting duration generally shorter than 12 weeks, usually within a range of 4 to 8 weeks ( Stadtreinigung Hamburg, 2019). In addition, there are differences in the composition of the input material fed into the composting process. Some facilities focus on garden waste while others are hybrid biogas and composting plants that predominantly run aerobic composting on the digestate from the anaerobic digestion process.

The behaviour of biodegradable plastics in the composting process has been studied in the literature. However, the duration and conditions of the composting process as well as the design and composition of the plastic vary. Ruggero *et al*., 2021 simulated an industrial composting process in the lab with 20 days of thermophilic composting (58°C) and 40 days of compost maturation (37°C) with mature compost as the composting medium. They tested a starch and polybutylene adipate terephthalate (PBAT) composite film, a PBAT based film and PLA pressed plate. Other studies focused on the design factors affecting the speed and degree of degradation. For example, micro fibrillated cellulose was found to increase the degradation of PLA composites while cellulose nanocrystals retard degradation ( Manzano *et al*., 2021). In addition to the concentration of fillers, the material thickness was also found to affect the degradation speed of PLA and PBS blends ( Tolga *et al*., 2020). Recently, researchers also studied the effect of treating compostable plastics in composting plants on the composting process itself. Cucina *et al*., 2021 researched the effects of the high loading of commercially available compostable plastic products in bio-waste (10 wt %) on compost quality. Certified compostable starch-based shopping bags and PLA-based cutlery degraded by 48 wt% and 15 wt% respectively after a combination of a pilot-scale mesophilic dry anaerobic digestion phase (35 days), active composting phase (15 days) and compost maturation phase (40 days). A final concentration of compostable plastic in compost was found to be around 18 wt%.

There is still a need to better understand the factors affecting disintegration rates in actual industrial composting conditions as well as its connection with lab-scale tests. This work thus assessed the degradation rate, measured by mass loss, of two biodegradable plastic prototypes developed for rigid (PLA-based) and soft packaging (PBS-based) respectively within the BIO-PLASTICS EUROPE research project in both lab-scale simulated ( DIN EN ISO 20200) and actual industrial composting conditions. The composting medium was characterized for comparison. The results outlined overall factors affecting the disintegration rate and explored the possible reasons for the differences in the material disintegration rate between the lab-scale and industrial-scale tests.

## Methods

### Biodegradable plastic test samples

The biodegradable plastic materials tested were a PLA-based biodegradable plastic blend (BPE-RP-PLA) developed for rigid packaging and a PBS-based biodegradable plastic blend (BPE-SP-PBS) developed for soft packaging. Table 1 lists the main characteristics of each blend and the sample thickness used in this study. 

**Table 1.** The main characteristics of the biodegradable plastic blends tested.

Blend code	BPE-RP-PLA	BPE-SP-PBS
**Target application**	Rigid packaging	Soft packaging
**Base polymer**	PLA	PBS
**Main Filler**	Calcium Silicate	Talc
**Ash content**	28.7%	9.7%
**Sample thickness**	1 mm plates	109±9 µm films

The blends are being developed in the framework of the H2020 Research Project BIO-PLASTICS EUROPE. The main filler in BPE-RP-PLA is calcium silicate with an overall ash content of 28.7%. Injection moulded plates of 1 mm thickness were used to represent rigid packaging. BPE-SP-PBS was tested in the form of films around 109 µm thick. The main filler is talc and the blend has an ash content of 9.7%. The thickness tested represents an approximation of the thickness the final products of the target application would have.

### Lab-scale industrial composting

The lab-scale test served as a baseline test for ideal industrial composting conditions. The lab-scale industrial composting test was carried out based on ISO 20200 and simulates the thermophilic industrial composting conditions. The composting medium was synthetic bio-waste composed of 40% sawdust, 30% rabbit feed, 10% fresh compost, 5% sucrose, 4% corn seed oil and 1% urea by dry mass. Brief descriptions of the material sources are given in *Extended data* ( Chong *et al*., 2022b). Fresh compost was taken from the Bützberg Biogas and Composting plant as inoculum. The water content was adjusted to 55 wt% at the start of the experiment.

For each reactor system, 1.1 kg of composting medium was mixed with around 10 g of plastic sample (~1 wt% sample loading) in polypropylene boxes with lids (dimensions 34 cm X 20 cm X 12.5 cm). The initial mass of the dry plastic samples was weighed and recorded before adding to the reactor. Each box had two holes of 5 mm diameter on the sides for aeration. For BPE-RP-PLA, samples cut into 2.5 cm X 2.5 cm pieces were used following ISO 20200. For BPE-SP-PBS films, individual pieces cut into 5 cm X 5 cm pieces were used instead to make them more manageable. The reactor systems were placed in a convection oven maintained at a temperature of 58°C throughout the experiment, simulating the thermophilic composting phase.

The reactor contents were weighed and the moisture content was replenished based on the schedule defined in ISO 20200 to ensure sufficient moisture content. In addition, the contents were gently mixed if instructed. The mass loss of the plastic samples was measured after 4, 8 and 12 weeks. Triplicate reactor systems were set up for each time point. In addition, 3 reactor systems without the plastic samples were set up as control.

At the end of the defined composting duration, the process was stopped by drying the reactor contents in the oven after removing the lid until constant weight is achieved. The mass before and after drying was used to estimate the final moisture content. The remaining plastic samples were recovered by sieving the reactor contents with a 2 mm mesh analytical sieve (RETSCH GmbH, Haan, Germany). The sample pieces were cleaned gently under running water and oven-dried at 58°C before final weighing. The initial and final weight was used to calculate the remaining mass using Equation 1. The remaining dried compost medium was then milled into powder and further analysed for pH, C/N ratio and volatile organic solids content using standard methods. Further details of the materials and methodologies used are described in *Extended data* ( Chong *et al*., 2022b).

$$Remaining\;mass\;(\%)=\frac{Mass_{final}>2\;mm}{Mass_{initial}}\times 100\%\;Equation 1$$

### On-field industrial composting test

To study the behaviour of biodegradable plastic samples in real-world conditions, the samples were subjected to industrial composting in the Bützberg Biogas and Composting Plant located in Tangstedt, Germany. The plant receives separately collected bio-waste from households in the region. Before undergoing biological treatment, the waste was shredded (< 8 cm), sieved and sent through magnetic separation to remove impurities. The plant then ran a dry anaerobic digestion process on the pre-treated biowaste for biogas production followed by in-vessel composting of the digestate material after anaerobic digestion. Hybrid fermentation chambers 24 m x 5 m x 4.5 m were used for the anaerobic digestion phase. For this study, the composting phase was carried out in the hybrid fermenters with aeration after the anaerobic digestion phase. The industrial composting outputs were finally sieved to produce compost for sale.

**Figure 1.**
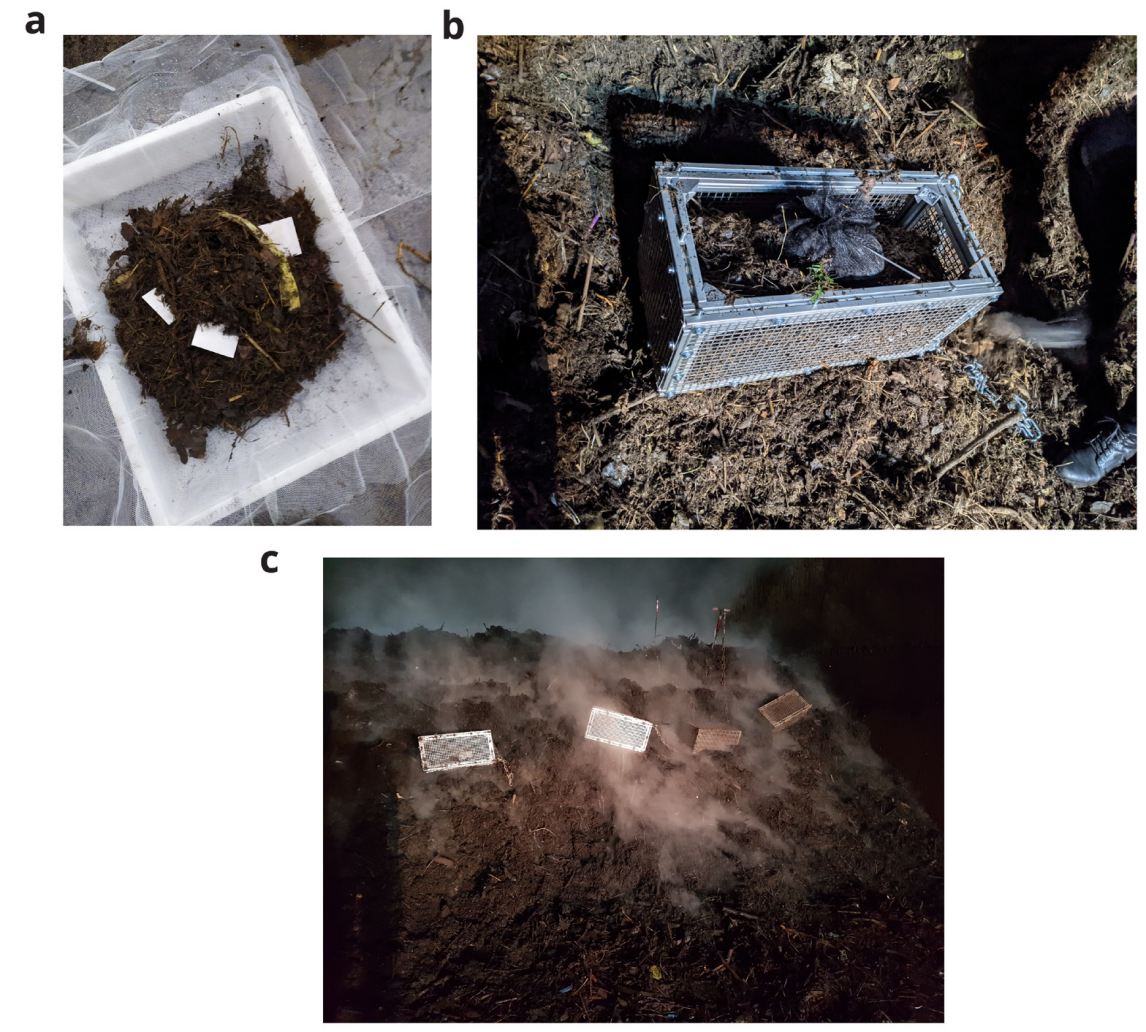
Experimental setup in the industrial composting plant. ( **a**) biodegradable plastic samples with digestate material in sample nets. ( **b**) sample nets in a metal cage. ( **c**) position of the cages in the composting pile.

For each test instance, around 10 g of sample and 1 kg of fresh digestate material from anaerobic digestion were mixed and placed into polyethylene terephthalate (PET) mesh bags with a mesh size of 1-2 mm. The initial mass of the dry plastic samples was weighed and recorded before adding to the PET mesh bags. This enabled recovery of sample pieces > 2mm at the end of the experiment for mass loss measurements. For BPE-RP-PLA, pieces of 5cm X 5cm were used according to DIN EN ISO 16929, the standard for pilot scale disintegration tests. For BPE-SP-PBS, individual film pieces of 10 cm X 10 cm were used. The bags were then sealed and placed into specially constructed metal sample cages (25 cm X 25 cm X 50 cm) with stainless steel grates with a mesh size of 10-12 mm. The cages were filled with more digestate material and placed in the middle of the composting pile together with two probes for temperature measurements. The experimental setup is shown in Figure 1. Each biodegradable plastic blend was tested in duplicate.

The exposure was carried out for three weeks, which is the composting duration for this batch of digestate material. After the first and second week, the composting pile was mixed in which the entire mass was transferred via a wheel loader from one hybrid fermentation chamber to another. In these phases, the sample cages were extracted, gently rotated to encourage mixing and reinserted into the composting pile. At the end of the composting process, the sample bags were extracted and dried in an oven at 105°C until constant weight. The plastic pieces were then extracted through sieving at 2 mm, washed and oven-dried at 58°C before final weighing. The initial and final weight was used to calculate the remaining mass using Equation 1. Samples of the composting medium were taken at the start and end of the experiment, oven-dried at 105°C to measure moisture content. The biomass was then ground into powder for further characterization of pH, C/N ratio and volatile solids content using standard methods described in *Extended data* ( Chong *et al*., 2022b).

## Results and discussion

### Disintegration rate in lab-scale industrial composting

**Figure 2.**
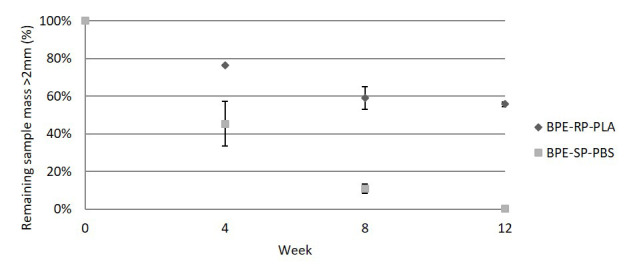
The remaining mass of samples in the lab-scale simulated industrial composting.

Figure 2 shows the average measured remaining mass of the samples larger than the 2 mm limit as defined by ISO 20200, as well as the standard deviation of the triplicate measurements. The remaining mass of BPE-SP-PBS is 45.4%, 10.9% and 0.3% by weeks 4, 8 and 12 respectively. BPE-SP-PBS in the current form will thus fulfil the 90% disintegration condition (10% remaining mass) set by ISO 20200 after a little over 8 weeks. In contrast, 55.7% of BPE-RP-PLA remained larger than 2 mm after 12 weeks with only a small difference between week 8 and week 12. BPE-RP-PLA in its current form thus does not fulfil the 90% disintegration condition. For both materials, the decrease in volatile solids of the composting medium (denoted R in ISO 20200) met the minimum requirement of the standard set at 30% from week 4 onward. The R-values and mass loss data are tabulated in *Underlying data* ( Chong *et al*., 2022a). The large standard deviation at week 4 for BPE-SP-PBS can be attributed to uneven contact of the films with the composting environment, as it was observed that the pieces above the composting medium experienced little disintegration. After week 4, the pieces reduced in size and thus could be evenly distributed within the composting medium. The deviations in mass loss of the other time points were within the 20% limit set by ISO 20200.

The lab-scale results indicate that BPE-SP-PBS of a thickness ≤ 109 µm would meet the disintegration requirement in an industrial composting plant as long as the residence time of thermophilic composting is longer than 8 weeks. On the other hand, BPE-RP-PLA in its current form would not disintegrate sufficiently in industrial composting plants with an active thermophilic composting time of fewer than 12 weeks.

### Disintegration rate in an industrial composting plant

**Figure 3.**
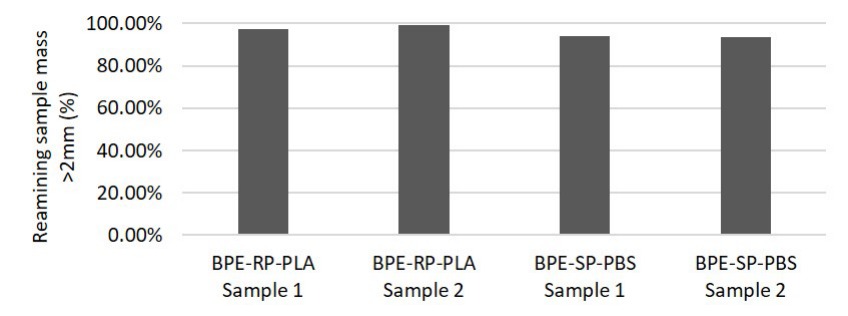
The remaining mass of samples after industrial composting on-field.

The degradation rates after exposure in an industrial composting plant are shown in Figure 3. The tests were conducted in duplicates. After 3 weeks of exposure to industrial composting of the digestate material from anaerobic digestion, the samples experienced only minor mass losses. For BPE-RP-PLA, the remaining mass was 97.2% and 99.5%. BPE-SP-PBS samples experienced slightly higher mass loss with 94.0% and 93.8% remaining mass.

The resulting mass loss after 3 weeks of industrial composting is much lower compared to the results from the lab-scale tests at week 4. This is most likely due to the differences in the compositing medium and conditions, which are further discussed in Section 3.3. Similar to the lab-scale test, BPE-SP-PBS experienced faster disintegration in general when compared to BPE-RP-PLA.

**Figure 4.**
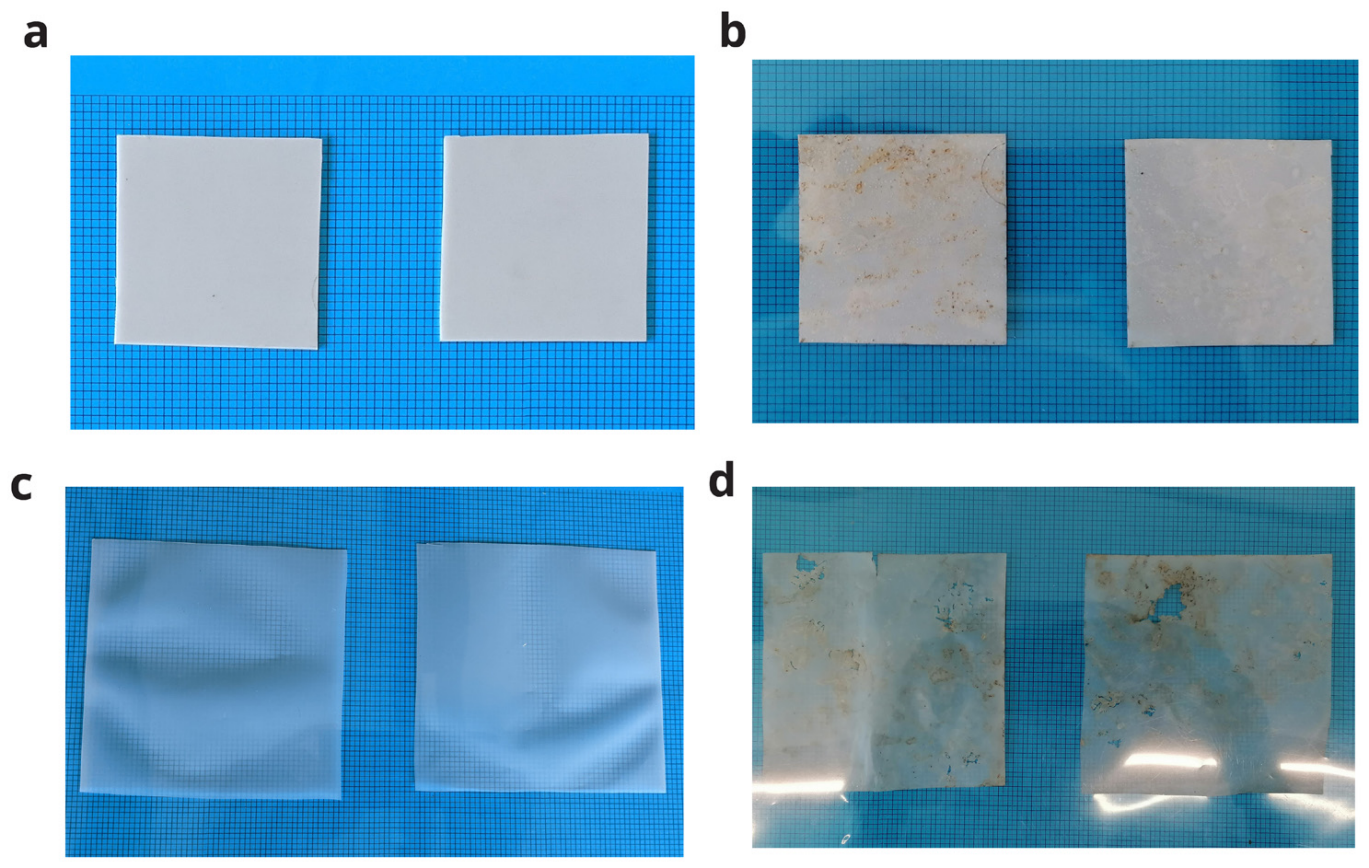
Samples before and after on-field industrial composting. ( **a**, **b**) BPE-RP-PLA. ( **c**, **d**) BPE-SP-PBS.

Although the reduction in mass is not significant, BPE-RP-PLA had significant visual changes including yellowing and unevenness on the surface. BPE-SP-PBS also experienced yellowing and warping with noticeable holes. Figure 4 depicts the material after industrial composting, cleaning and drying. In addition, all samples became more brittle and prone to fragmentation. This signifies a structural degradation of the polymer matrix.

The results indicate that the blends in the current form are not suitable for treatment in the industrial composting process where the test was carried out. Although degradation of the material was observed, the disintegration rate is very low within the active composting duration of the plant.

### Differences in composting conditions between the lab-scale and on-field tests

The lab-scale test based on ISO 20200 simulates a controlled thermophilic environment with a well-defined composting medium with small particle sizes (95% < 1 cm). On the other hand, the digestate material with which industrial composting was carried out had a larger and broader particle size distribution (shredded to ≤ 8cm). The composition would also change seasonally depending on separately collected biowaste from households. The measured pH, moisture content, volatile solids content and C/N ratio of the composting medium in both tests are listed in Table 2. The standard deviation of the total volatile solid measurements reflects the heterogeneity of the on-field composting medium, which had higher variation compared to the composting medium used in the lab.

**Table 2.** Lab-scale and on-field composting medium at the start of the experiment.

Test type	Lab-scale	On-field
**Brief ** **description**	A well-defined mix of materials based on ISO 20200.	A mixture of food and garden waste after dry anaerobic digestion. The composition depends on separately collected biowaste in the region.
**Particle size**	95% < 1cm	≤ 8cm
**Total dry solids ^1^**	45.0% (Start) ^4^ 26.6%±1.0% (End) ^5^	37.1%±5.0% (Start) 47.6%±1.9% (End)
**Total volatile ** **solids ^2^**	90.6%±0.6%	37.8%±2.6%
**pH ^3^**	5.8±0.1	6.9±0.1
**C/N ratio ^3^**	33±2	19±1

1 Based on wet weight. 2 Based on total dry solids. 3 Measured on oven-dried and ground samples. 4 The moisture content was adjusted at the start of the experiment and thus not measured. 5 After four weeks of composting BPE-SP-PBS.

The average volatile solid content of the lab-scale composting medium was higher compared to the on-field medium. Since the on-field medium stems from biowaste, it is expected that the inorganic content, i.e. sand and dust, will be higher due to the mixed collection of food and garden waste. The pH of the lab-scale composting medium was slightly acidic while the pH of the on-field medium was neutral; both in the accepted range for bacteria and fungi ( Chen *et al*., 2011). The C/N ratios of the lab-scale and on-field composting medium were close to the optimal for composting, quoted at 25 to 30 ( Kumar *et al*., 2010). Generally, it could be argued that the lab-scale composting medium with its smaller particle sizes and higher organic solids content is more conducive to disintegration as it facilitates a better sample-medium contact and higher biological activity. This could explain the better disintegration rates of the samples in the lab. In addition, the composting medium in the lab-scale test is mixed more frequently (based on the schedule in ISO 20200) compared to the on-field composting medium (once a week).

**Figure 5.**
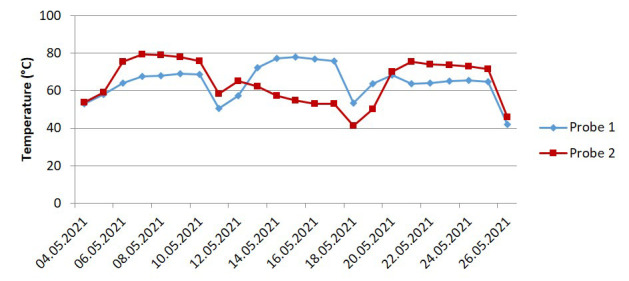
The temperature profile of the on-field industrial composting ( Bützberg Biogas and Composting Plant, 2021).

Moreover, the temperature profiles of the processes differed. In the lab-scale experiment, the temperature was kept constant at 58°C via a convection oven. In contrast, the temperature of the on-field composting medium depended on self-heating and went through multiple cycles corresponding to the mixing schedules. The profiles of the temperature measured by the two probes are shown in Figure 5. The two dips in the middle correspond to the mixing phase, where the composting chambers are open and the composting medium is transferred from one chamber to another to facilitate mixing. The temperature ranged from 40°C to 80°C with an approximate median of 60°C. In both cases, the temperatures were well within the thermophilic range. Indicated by the measurements at the start and end of the experiments, the lab-scale test experienced wetter environments compared to the on-field test as a result of the methodology laid out in ISO 20200. A wetter environment might facilitate better disintegration of PLA and PBS. The disintegration of PLA initially relies on chemical hydrolysis to break down long-chain polymers ( Mochizuki & Hirami, 1997) and thus benefits from higher humidity ( Karamanlioglu *et al*., 2017). The degradation of PBS relies on enzymatic activity on the surface ( Su *et al*., 2019) as well as hydrolysis ( Muthuraj *et al*., 2015) and thus would also benefit from increased moisture.

### Feasibility of treating compostable plastic packaging in industrial composting plants

This study indicates that both blends in their current form do not meet the 90% disintegration requirement for compostable packaging according to EN 13432 after exposure to the process at the Bützberg plant. The disintegration rates might be higher if the samples are not contained in sample nets and metal cages and thus more susceptible to mechanical stresses during the composting process, i.e. during mixing. However, due to the very low disintegration rates observed, i.e. all samples were lower than 7%, it is unlikely that the disintegration rates will improve above the 90% disintegration rate required by ISO 20200 and EN 13432.

The disintegration of BPE-SP-PBS in other industrial composting plants might meet the requirement if the thermophilic composting phase is longer than 8 weeks with fresh biowaste as the composting medium, as indicated by the lab-scale test results. However, the thermophilic composting phase of most industrial processes does not exceed 3 weeks ( Ruggero *et al*., 2021). For BPE-RP-PLA, the thermophilic composting phase would need to be longer than 12 weeks to achieve sufficient degradation according to the lab-scale test results.

The conditions defined for certified compostable packaging, for example, those with their disintegration tested according to the pilot-scale disintegration test according to ISO 16929, are 4 weeks of composting above 55°C, 4 weeks of composting above 50°C and 4 weeks of composting below 45°C using fresh biowaste as inputs. These conditions also deviate from those in Bützburg and similar plants, and thus the disintegration rate is expected to be low.

An important determining factor for disintegration is the shape and form of the biodegradable packaging articles, i.e. Tolga *et al*., (2020) found that the degree of disintegration linearly correlated with the thickness of PLA/PBS blends. Especially for rigid packaging, the thickness presents an extra obstacle. Extra pre-processing steps, i.e. shredding, would likely be needed if the disintegration of biodegradable plastics in industrial composting plants is to be increased. Further research on the effects of differing composting medium compositions, organic solids content, and particle size distributions are needed to understand the scenarios in which the disintegration rates will meet requirements.

It should be noted that the disintegration rate with a scope above 2 mm is not sufficient when considering the feasibility of treating compostable plastic in industrial composting plants. The behaviour and ultimate biodegradation of the smaller compostable plastic particles in the environments in which they might enter, i.e. agricultural or garden soil, as well as their toxicity, should be determined ( Fojt *et al*., 2020; Karamanlioglu & Alkan, 2020). Lastly, integration with the wider waste management system needs to be considered. For one, the motivation of the industrial composting plants to sanitize biowaste and produce compost might not align with the treatment of compostable plastics, which might complicate the process without added benefit to the plant operator.

## Conclusion

Heeding the resistance from some waste management authorities to treat compostable plastics in composting plants, the behaviour of biodegradable and compostable plastics under actual industrial composting conditions should be thoroughly examined to assess their environmental impact. Therefore, this study investigated the disintegration rate of a PLA blend and a PBS blend designed for rigid and soft packaging respectively under simulated lab-scale and on-site industrial composting conditions. While under simulated industrial conditions according to ISO 20200, the remaining mass of BPE-RP-PLA and BPE-SP-PBS samples were 76.4% and 45.4% respectively after 4 weeks and 55.7% and 0.3% after 12 weeks. On the contrary, the degree of disintegration under on-site conditions was significantly lower with remaining masses at least 97.2% for the BPE-RP-PLA samples and 93.8% for the BPE-SP-PBS samples after 3 weeks of thermophilic composting. Apart from the thickness of the plastic samples and the duration of the thermophilic composting phase, composting conditions such as substrate composition and particle size could be major influencing factors.

Although BPE-SP-PBS samples in their current thickness almost disintegrated entirely after 12 weeks, a minimum thermophilic composting duration of 8 weeks would be necessary to reach the 90% disintegration (10% remaining mass) required by EN 13432, which exceeds the thermophilic phase of most composting plants. Biological waste treatment plants including composting plants are generally designed to treat and sanitize biological waste and are not optimized to treat compostable plastics, especially concerning the retention time. Henceforth, realistic certification requirements that reflect differing retention times and substrate composition which occur in real-life applications should be considered. Furthermore, systemic integration considering the biowaste value chain and the motivations of industrial composting plants is critical.

## Data availability

### Underlying data

B2Share: Underlying data_Lab-scale and on-field industrial composting of biodegradable plastic blends for packaging_Chong *et al*., 2022

http://doi.org/10.23728/b2share.96956d1bae7d40cb80e0c6b4dfa0b30f ( Chong *et al*., 2022a)

This project contains the following underlying data:

- CN ratio_Industrial test.xlsx
- CN ratio_lab-scale.xlsx
- Data list.docx
- Moisture content.xlsx
- pH.xlsx
- Remaining mass industrial test.xlsx
- Remaining mass lab-scale_BPE-RP-PLA.xlsx
- Remaining mass lab-scale_BPE-SP-PBS.xlsx
- R value lab-scale test reactors_BPE-RP-PLA.xlsx
- R value lab-scale test reactors_BPE-SP-PBS.xlsx
- Total volatile solids.xlsx

### Extended data

B2Share: Extended data_Lab-scale and on-field industrial composting of biodegradable plastic blends for packaging_Chong *et al*., 2022

http://doi.org/10.23728/b2share.5356b112d6bd424aa79b10d397ee1b81 ( Chong *et al*., 2022b)

This project contains the following extended data:

- Extended data_Methodology.docx

Data are available under the terms of the Creative Commons Attribution 4.0 International license (CC-BY 4.0).
