# Lab-scale and on-field industrial composting of biodegradable plastic blends for packaging

**DOI:** 10.12688/openreseurope.14893.2

**Published:** 2023-09-13

**Authors:** Zhi Kai Chong, Alexander Hofmann, Marie Haye, Sharon Wilson, Ihsanullah Sohoo, Ayah Alassali, Kerstin Kuchta

**Affiliations:** 1 Circular Resource Engineering and Management (CREM), Hamburg University of Technology, Hamburg, 21073, Germany; 2 Department of Energy and Environmental Engineering (GEn), Institut National des Sciences Appliquées de Lyon, Villeurbanne, 69100, France

**Keywords:** Biodegradable plastic, compostable plastic, compostable packaging, industrial composting, polylactic acid, polybutylene succinate

## Abstract

**Background**: The acceptance of compostable plastic packaging in industrial composting plants is not universal despite available certification due to the persistence of plastic residues after composting. To better understand this discrepancy, this study compared the disintegration rates of two blends designed for rigid packaging (polylactic acid based) and soft packaging (polybutylene succinate based) in lab-scale composting tests and in an industrial composting plant.

**Methods**: A lab-scale composting test was conducted in triplicates according to ISO 20200 for 4, 8 and 12 weeks to check the disintegration potential of the blends. Duplicate test material were then exposed in the compost pile of an industrial composting plant for a duration of 3 weeks and compared with a supplementary lab-scale test of the same duration.

**Results**: The rigid packaging samples (1 mm thickness) retained on average 76.4%, 59.0% and 55.7% of its mass after 4, 8 and 12 weeks respectively in the lab-scale. In the plant, the remaining mass was 97.2% and 99.5%, much higher compared to the average of 68.9% after 3 weeks in the supplementary lab-scale test. The soft packaging samples (109±9 µm sample thickness) retained on average 45.4%, 10.9% and 0.3% of its mass after 4, 8 and 12 weeks respectively in the lab-scale. In the plant, a high remaining mass was also observed (94.0% and 93.8%). The supplementary lab-scale test showed similar remaining mass but higher fragmentation after 3 weeks.

**Conclusions**: The results show that the samples achieved significant disintegration in the lab-scale but not in the plant. The difference between the tests that might further contribute to the differing degradation rates is the composition and heterogeneity of the composting substrate. Therefore, the substrate composition and thermophilic composting duration of individual plants are important considerations to determine the suitability of treating compostable plastic in real-world conditions.

## Plain language summary

There is resistance from industrial composting plants to the treatment of compostable biodegradable plastics. This study aims to compare the disintegration rates of two new biodegradable plastic blends developed for rigid and soft packaging applications in controlled industrial composting conditions in the lab as well as under actual industrial composting conditions in a composting plant. Results show significant disintegration of the material in the lab-scale up to 12 weeks. However, there were notable differences in the degree of disintegration of the samples in the lab compared to real-world conditions after 3 weeks. The 1 mm thick polylactic acid-based blend for rigid packaging experienced much higher disintegration in the lab with 68.9% remaining mass after 3 weeks compared to 97.2% remaining mass in the industrial composting plant. The 109 µm thick polybutylene succinate-based blend for soft packaging had similar remaining masses comparing the lab and on-field tests. However, the lab-test showed higher fragmentation after 3 weeks. The characteristics of the organic waste inputs are potential causes of the lower disintegration rates. The study highlights the need to better understand the real-world industrial composting conditions and their variations when evaluating composting as a treatment method for biodegradable plastics.

## Introduction

The prevalence of plastic waste and micro-plastic in the environment has spurred research and development of alternatives such as biodegradable plastics. Biodegradable plastics include polymers that can be broken down and mineralized by microbial action. Commonly available bio-based biodegradable plastics include polylactic acid (PLA) and polybutylene succinate (PBS) ( IfBB, 2020). They can be produced from both fossil-based and bio-based resources. It is important to note the difference between the terms biodegradation and disintegration. In EN 13432, biodegradability is to be proved via the decomposition of carbon in the plastic material ultimately into carbon dioxide. Disintegration on the other hand, refers to the fragmentation into very small pieces, i.e. measured by the loss of mass above a size threshold, > 2 mm in the case of EN 13432. Compostable plastics are a subcategory of biodegradable plastics that disintegrate significantly in specific composting conditions, but not necessarily in general environmental conditions on land or sea. Thus, they need to be kept in controlled closed systems.

Industrial compostable products are designed to disintegrate within a reasonable period under industrial composting conditions. The major characteristic of industrial composting is the ability to achieve thermophilic temperatures ( Barrena *et al*., 2014; Sundberg *et al*., 2004), in the range from 55°C to 75°C. Composting might be an alternative for the treatment of plastic waste, for example when mechanical recycling is difficult, such as in the case of multilayer packaging ( de Mello Soares *et al*., 2022; Trinh *et al*., 2021). However, industrial composting primarily aims to treat and stabilize organic waste and produce compost, an agricultural substrate. Thus, the feasibility of the treatment of biodegradable plastics within these plants is not a given.

For instance, there is no widespread acceptance and treatment of compostable plastics in industrial composting plants in Germany ( ANS e.V. (ANS) *et al*., 2019). One of the major issues quoted is the insufficient decomposition of biodegradable plastic products within the normal operating conditions of the plants. This occurs even with products certified as industrially compostable via international standards such as EN 13432. The reason is the wide range of different conditions in which industrial composting operators run their plants, which are sometimes quite distinct from the compostability certification conditions ( Di Bartolo *et al*., 2021; Hann *et al*., 2020). The disintegration condition in EN 13432 requires that the product disintegrates under industrial composting conditions and leaves behind less than 10% of the material (> 2 mm) by mass after a maximum duration of 12 weeks in a “controlled pilot-scale test” or in industrial composting plants. The industrial composting plants in Germany on the other hand, have an active composting duration usually within a range of 4 to 8 weeks ( Stadtreinigung Hamburg, 2019). In addition, there are differences in the composition of the input material fed into the composting process. Some facilities focus on garden waste while others are hybrid biogas and composting plants that predominantly run aerobic composting on the digestate from the anaerobic digestion process.

The behavior of biodegradable plastics in the composting process has been studied in literature in the lab and pilot scale. However, the duration and conditions of the composting process as well as the design and composition of the plastic vary. Ruggero *et al*., 2021 simulated an industrial composting process in the lab with 20 days of thermophilic composting (58°C) and 40 days of compost maturation (37°C) with mature compost as the composting medium. They tested a starch and polybutylene adipate terephthalate (PBAT) composite film (50 µm thick), a PBAT based film (90 µm thick) and PLA pressed plate (500 µm). It was found that the starch and PBAT film underwent the highest mass loss (45%), whilst the PBAT film and PLA plate underwent low mass losses, of 8% and 3% respectively. The authors attributed the low mass loss of PLA plates to the short thermophilic phase as well as the higher thickness compared to other studies. Other studies focused on the design factors affecting the speed and degree of degradation. For example, microfibrillated cellulose was found to increase the degradation of PLA composites while cellulose nanocrystals retard degradation ( Manzano *et al*., 2021). The material thickness was also found to correlate with the disintegration speed of PLA and PBS blends ( Tolga *et al*., 2020). In the same study, the disintegration rate of a 7:3 PLA:PBS blend was in a range of 25% to 35% after 12 weeks, depending on the thickness from 1 mm to 2 mm. Chalk was found to promote degradation possibly through facilitating water penetration while talc had the opposite effect. Researchers also studied the effect of treating compostable plastics in composting plants on the composting process itself. Cucina *et al*., 2021 researched the effects of the high loading of commercially available compostable plastic products in organic waste (10 wt%) on compost quality. In the mentioned study, certified compostable starch-based shopping bags and PLA-based cutlery degraded by 48 wt% and 15 wt% respectively after a combination of a pilot-scale mesophilic dry anaerobic digestion phase (35 days), active composting phase (15 days) and compost maturation phase (40 days). A final concentration of compostable plastic in compost was found to be around 18 wt%. Gadaleta *et al.* in turn found no effect of a 2% loading of cellulose acetate films on lab-scale anaerobic digestion and composting processes but noted also the presence of non-degraded material at the end of the process ( Gadaleta *et al.*, 2022).

In a field study, 0.3 mm PLA rigid films were exposed in static compost piles comprising garden and food waste at a temperature range of 52°C to 59°C ( Sikorska *et al.*, 2015). After 21 days, a decrease in the average molecular weight of the material from 105000 (Da) to 55000 (Da) was observed without significant mass loss. Another study exposed commercial PLA bottles to a mixture of cow manure, feed and wood shavings in a compost pile at 65°C ( Kale *et al.*, 2007). Complete disintegration within 30 days was observed visually. In addition, the molecular weight dropped from an initial 230000 Da to below 50000 Da within 10 days. Similarly, a composting test in a static aerated compost pile for PLA/PHA mulch films reported high disintegration, with a 92% loss of surface area after two weeks ( Sintim *et al.*, 2019).

Overall, the availability of published papers about full-scale composting tests in industrial plants is limited with varying outcomes. A study exposed various commercially available products made from compostable plastics to source separated municipal organic waste in an industrial composting plant for a total of 22 days with a temperature range of 30°C to 70°C throughout the run ( van der Zee & Molenveld, 2020). A wide range of disintegration from 6% to 100% was obtained for different types of biodegradable polymers in different forms with a size cutoff of 2 mm. For PLA-based rigid materials, duplicate samples of plant pot cuttings achieved almost 100% disintegration, while the duplicate coffee capsule samples had a disintegration of 9% and 41% respectively. A research group exposed PLA/PBAT blend films from 0.02 mm to 0.1 mm thickness in “KNEER” container composting systems with an average temperature of 60°C ( Musioł *et al.*, 2018). Though not explicitly measured, it was observed that significant disintegration only started after 21 days of incubation. In another study, certified compostable PLA tableware exposed to in-vessel composting using a mixture of bio-solids, food waste and woody material for an active phase of up to 14 days at 60°C (with recirculation of sample pieces >2 cm) and further curing for around 4 months ( Zhang *et al.*, 2017). Complete disintegration was reported for all samples with a size cut-off of 3.2 mm.

Chemically, the disintegration of PLA in industrial composting conditions can be understood through two mechanisms: the abiotic hydrolysis of the polymer chains in the presence of water and heightened temperatures resulting in shorter chains as well as microbiological enzymatic attack on the shorter chains ( Karamanlioglu *et al.*, 2017). Above the glass transition temperature, higher chain mobility facilitates chemical and biological degradation ( Karamanlioglu & Robson, 2013). Hydrolysis of PLA in aqueous media is accelerated with increased temperature, especially above the glass transition temperature reported around 57°C to 61°C ( Rodriguez *et al.*, 2016). In addition, the mass loss trend with respect to composting time was reported to follow an S-shape curve, with an incubation phase at the beginning in which a rapid decrease in molecular weight was observed with no mass losses followed by a rapid decrease in mass after the incubation period. A lag phase was also observed for biodegradable plastics in some studies at the start of the composting process ( Anstey *et al.*, 2014; Zhao *et al.*, 2005). This trend was also observed in industrial composting conditions for PLA/PBS blends ( Tolga *et al.*, 2020).

Both acidic and basic pH can catalyze the abiotic hydrolysis of polyesters ( Woodard & Grunlan, 2018). There are studies that reported that basic media (pH 12 to >13) facilitated the surface erosion of polylactic acid ( von Burkersroda *et al.*, 2002) and the weight loss of poly(butylene adipate-co-butylene furandicarboxylate)s (PBAFs) and poly(butylene succinate-co-butylene furandicarboxylate)s (PBSF) ( Peng *et al.*, 2017). Although there are various studies reporting the ability of microorganisms to biodegrade PLA ( Al Hosni *et al.*, 2019; Kawai, 2010), there is still a debate on the effect of microorganisms compared to abiotic factors in terms of macro-scale material disintegration. PBS is also susceptible to both abiotic hydrolytic degradation ( Lindström *et al.*, 2004) and enzymatic degradation ( Xu & Guo, 2010). Therefore, there is still a need to better understand the factors affecting disintegration rates in actual industrial composting conditions as well as its connection to controlled lab-scale tests. This work thus assessed the degradation rate, measured by mass loss, of two biodegradable plastic prototypes developed for rigid (PLA-based) and soft packaging (PBS-based) respectively within the BIO-PLASTICS EUROPE research project in both lab-scale simulated ( DIN EN ISO 20200) and actual industrial composting conditions. The composting medium was characterized for comparison. The results outlined overall factors affecting the disintegration rate and explored the possible reasons for the differences in the material disintegration rate between the lab-scale and industrial-scale tests. In addition, the composting duration factor was tested in the lab-scale to draw conclusions about the impact of duration variation in different composting facilities.

## Methods

### Biodegradable plastic test samples

The biodegradable plastic materials tested were a PLA-based biodegradable plastic blend (BPE-RP-PLA) developed for rigid packaging and a PBS-based biodegradable plastic blend (BPE-SP-PBS) developed for soft packaging. Table 1 lists the main characteristics of each blend and the sample thickness used in this study.

**Table 1.** The main characteristics of the biodegradable plastic blends tested.

Blend code	BPE-RP-PLA	BPE-SP-PBS
**Target application**	Rigid packaging	Soft packaging
**Base polymer**	PLA	PBS
**Main Filler**	Calcium Silicate	Talc
**Ash content**	28.7%	9.7%
**Sample thickness**	1 mm plates	109±9 µm films ^[1]^
**Specific surface area**	14 cm ^2^/g	130±11 cm ^2^/g ^[1]^
**Glass transition temperature (T _g_)**	59°C ^[3]^	Around -30°C ^[2]^
**Mass average molecular weight**	237000 g/mol ^[3]^	N/A

[1] ± denotes standard deviation; [2] Typical range for PBS from ( Su *et al.*, 2019; Wang *et al.*, 2019); [3] Data from the producer;

The blends were developed in the framework of the H2020 Research Project BIO-PLASTICS EUROPE. The main filler in BPE-RP-PLA is calcium silicate with an overall ash content of 28.7%. Injection molded plates of 1 mm thickness were used to represent rigid packaging. On the other hand, BPE-SP-PBS was tested in the form of films, with a thickness of 109 µm. The main filler is talc and the blend has an ash content of 9.7%. The thickness tested represents an approximation of the thickness that the final products of the target application would have.

### Lab-scale industrial composting

The lab-scale test served as a baseline test for ideal industrial composting conditions as well as to check the disintegration potential of the blends. The lab-scale test was carried out based on ISO 20200, which simulates the thermophilic industrial composting conditions. The composting medium was synthetic bio-waste composed of 40% sawdust, 30% rabbit feed, 10% fresh compost, 5% sucrose, 4% corn seed oil and 1% urea by dry mass. Brief descriptions of the material sources are given in *Extended data* ( Chong *et al*., 2022b). Fresh compost was taken from the Bützberg Biogas and Composting plant as inoculum. The water content was adjusted to 55 wt% at the start of the experiment.

For each reactor system, 1.1 kg of composting medium was mixed with around 10 g of plastic sample (~1 wt% sample loading) in polypropylene boxes with lids (dimensions 34 cm X 20 cm X 12.5 cm). The initial mass of the dry plastic samples was weighed and recorded before adding to the reactor. Each box had two holes of 5 mm diameter on the sides for aeration. For BPE-RP-PLA, samples cut into 2.5 cm X 2.5 cm pieces were used following ISO 20200. For BPE-SP-PBS films, individual pieces cut into 5 cm X 5 cm were used instead to make them more manageable. The reactor systems were placed in a convection oven maintained at a temperature of 58°C throughout the experiment, simulating the thermophilic composting phase.

The reactor contents were weighed and the moisture content was replenished based on the schedule defined in ISO 20200 to ensure sufficient moisture content. The contents were gently mixed in specific intervals according the schedule. The mass loss of the plastic samples was measured after 4, 8 and 12 weeks. Triplicate reactor systems were set up for each time point. In addition, 3 reactor systems without the plastic samples were set up as control.

At the end of the defined composting duration, the process was terminated by drying the reactor contents in the oven after removing the lid until constant weight was achieved. The mass before and after drying was used to estimate the final moisture content. The remaining plastic samples were recovered by sieving the reactor contents with a 2 mm mesh analytical sieve (RETSCH GmbH, Haan, Germany). The sample pieces were cleaned gently under running water and oven-dried at 58°C before final weighing. The initial and final weight was used to calculate the remaining mass using Equation 1. The remaining dried compost medium was then milled into powder and further analyzed for pH, C/N ratio and volatile organic solids content using standard methods. Further details of the materials and methodologies used are described in *Extended data* ( Chong *et al*., 2022b).

$$Remaining\;mass\;(\%)=\frac{Mass_{final}>2\;mm}{Mass_{initial}}\times 100\%\;Equation 1$$

### On-field industrial composting test

To study the behavior of biodegradable plastic samples in real-world conditions, the samples were subjected to industrial composting in the Bützberg Biogas and Composting Plant located in Tangstedt, Germany. The plant receives separately collected bio-waste from households in the region. Before undergoing biological treatment, the waste was shredded (< 8 cm), sieved and sent through magnetic separation to remove impurities. The plant then ran a dry anaerobic digestion process on the pre-treated organic waste for biogas production followed by in-vessel composting of the digestate material. Hybrid fermentation chambers 24 m x 5 m x 4.5 m were used for the anaerobic digestion phase. For this study, the composting phase was carried out in the hybrid fermenters with aeration after the anaerobic digestion phase. The industrial composting outputs were finally sieved to produce compost for sale.

**Figure 1.**
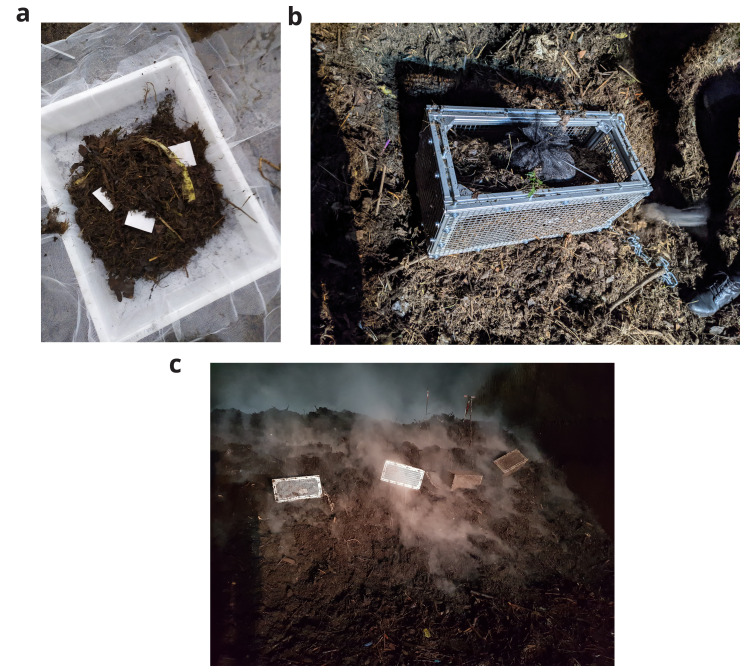
Experimental setup in the industrial composting plant.. ( **a**) biodegradable plastic samples with digestate material in sample nets. ( **b**) sample nets in a metal cage. ( **c**) position of the cages in the composting pile. The exposure was carried out for three weeks, which is the composting duration for this batch of digestate material. After the first and second week, the composting pile was mixed in which the entire mass was transferred via a wheel loader from one hybrid fermentation chamber to another. In these phases, the sample cages were extracted, gently rotated to encourage mixing and reinserted into the composting pile. At the end of the composting process, the sample bags were extracted and dried in an oven at 105°C until a constant weight was reached. The plastic pieces were then extracted through sieving at 2 mm, washed and oven-dried at 58°C before final weighing. The initial and final weight was used to calculate the remaining mass using Equation 1. Samples of the composting medium were taken at the start and end of the experiment, oven-dried at 105°C to measure moisture content. The biomass was then ground into powder for further characterization of pH, C/N ratio and volatile solids content using standard methods described in *Extended data* ( Chong *et al*., 2022b).

For each test instance, around 10 g of sample and 1 kg of fresh digestate material from anaerobic digestion were mixed and placed into polyethylene terephthalate (PET) mesh bags with a mesh size of 1–2 mm. The initial mass of the dry plastic samples was recorded before adding to the PET mesh bags. This enabled recovery of sample pieces > 2 mm at the end of the experiment for mass loss measurements. For BPE-RP-PLA, pieces of 5 cm X 5 cm were used according to DIN EN ISO 16929, the standard for pilot scale disintegration tests. For BPE-SP-PBS, individual film pieces of 10 cm X 10 cm were used. The bags were then sealed and placed into specially constructed metal sample cages (25 cm X 25 cm X 50 cm) with stainless steel grates with a mesh size of 10–12 mm. The cages were filled with more digestate material and placed in the middle of the composting pile together with two probes for temperature measurements. The experimental setup is shown in Figure 1. Each biodegradable plastic blend was tested in duplicate.

To facilitate direct comparison, a second lab-scale tests based on ISO 20200 was conducted using an exposure duration of three weeks as well as identical sample dimensions used for the on-field test. This test was done in triplicate.

## Results and discussion

### Disintegration rate in lab-scale industrial composting

**Figure 2.**
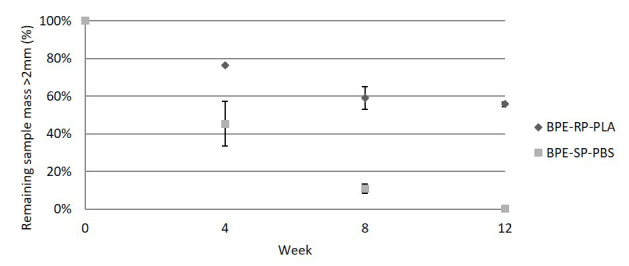
The remaining mass of samples in the lab-scale simulated industrial composting. Error bars denote standard deviation.

Figure 2 shows the average measured remaining mass of the samples larger than the 2 mm limit as defined by ISO 20200, as well as the standard deviation of the triplicate measurements. The remaining mass of BPE-SP-PBS is 45.4%, 10.9% and 0.3% by weeks 4, 8 and 12 respectively. BPE-SP-PBS in the current form will thus fulfil the 90% disintegration condition (10% remaining mass) set by ISO 20200 after a little over 8 weeks. In contrast, 55.7% of BPE-RP-PLA remained larger than 2 mm after 12 weeks with only a small decrease between week 8 and week 12. BPE-RP-PLA in its current form thus does not fulfil the 90% disintegration condition based on ISO 20200. For both materials, the decrease in volatile solids of the composting medium (denoted R in ISO 20200) met the minimum requirement of the standard set at 30% from week 4 onward. The R-values and mass loss data are tabulated in *Underlying data* ( Chong *et al*., 2022a). The large standard deviation at week 4 for BPE-SP-PBS can be attributed to uneven contact of the films with the composting medium, as it was observed that the pieces above the composting medium experienced little disintegration. After week 4, the pieces reduced in size and thus could be evenly distributed within the composting medium.

The lab-scale results indicate that BPE-SP-PBS of a thickness ≤ 109 µm would meet the disintegration requirement in an industrial composting plant as long as the residence time of thermophilic composting is longer than 8 weeks. On the other hand, BPE-RP-PLA in its current form would not disintegrate sufficiently in industrial composting plants with an active thermophilic composting time of fewer than 12 weeks. A reduction in thickness to achieve a higher surface area to volume ratio or changes to the blend composition are potential approaches to increase the disintegration rate from the material design point of view. In addition, it should be noted that the composting temperature of 58°C is slightly lower than the glass transition temperature of 59°C for BPE-RP-PLA. An increase in the composting temperature should also facilitate faster disintegration.

Comparing PLA and PBS, PLA/PBS blends with a higher ratio of PLA was reported to achieve higher disintegration rates in industrial composting conditions ( Tolga *et al.*, 2020). The authors attributed this to a higher autocatalytic degradation phenomenon in PLA. The same study also showed that thickness has a strong effect on the disintegration speed. A lower abiotic degradation rate of PBS compared to PLA in an alkali solution of pH 13 at 37°C was proposed from the results of a study ( Wang *et al.*, 2016). In addition, the filler content and type differed for the two blends. Talc used in BPE-SP-PBS was reported in a paper to retard disintegration of PLA/PBS blends ( Tolga *et al.*, 2020). The effect of calcium silicate on the biodegradability of bioplastic was not yet directly studied in research. The much lower thickness and thus much higher specific surface area of the BPE-SP-PBS (130 cm ^2^/g) samples likely contributed to a higher disintegration rate compared to the thicker BPE-RP-PLA (14 cm ^2^/g) in the timeframe studied.

### Disintegration rate in an industrial composting plant

**Figure 3.**
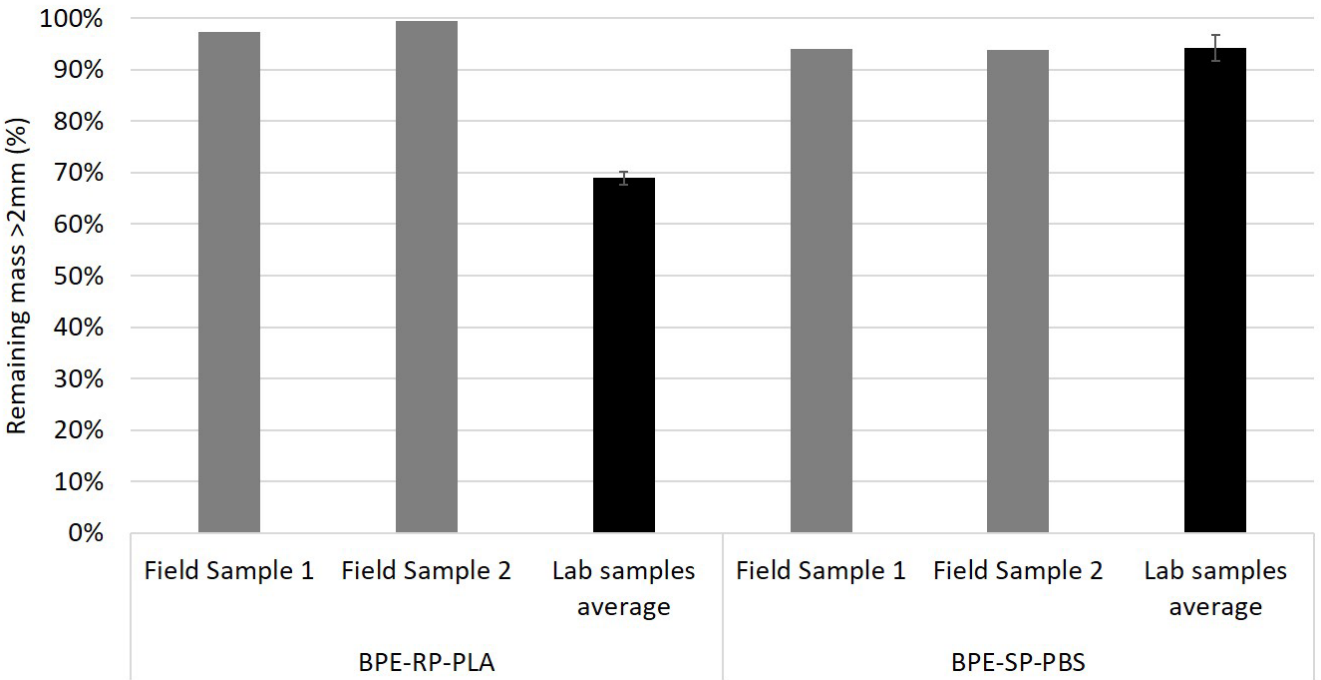
The remaining mass of samples after industrial composting on-field (in grey) in comparison to the lab-scale composting test (in black) for a duration of 3 weeks. Error bars denote standard deviation.

The degradation rates after exposure in an industrial composting plant are shown in Figure 3. In addition, the results from the second round of lab-scale test using identical sample dimensions and exposure duration are shown for comparison. After 3 weeks of exposure to industrial composting of the digestate material from anaerobic digestion, the plastic samples experienced only minor mass losses. For BPE-RP-PLA, the remaining mass was 97.2% and 99.5%. BPE-SP-PBS samples experienced slightly higher mass loss with remaining mass of 94.0% and 93.8%. After 3 weeks of exposure based on ISO 20200 in the lab, the remaining mass of BPE-RP-PLA and BPE-SP-PBS were on average 68.9% and 94.2%, respectively.

**Figure 4.**
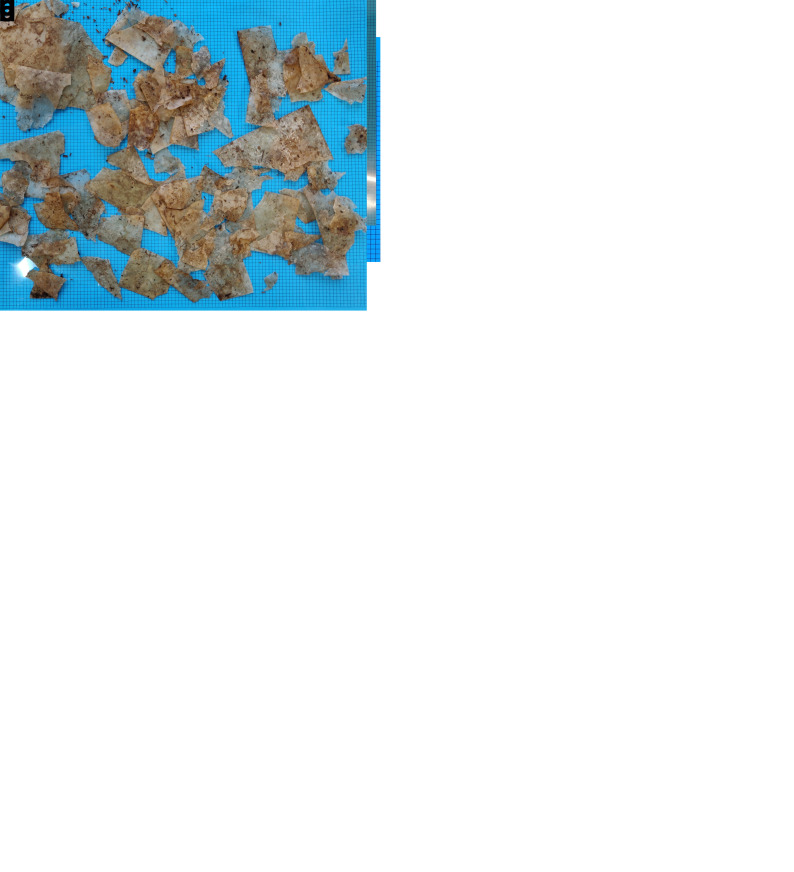
Samples before and after industrial composting. ( **a**, **b**) BPE-RP-PLA before and after 3 weeks of on-field composting. ( **c**, **d**) BPE-SP-PBS before and after 3 weeks of on-field composting. ( **e**) BPE-SP-PBS samples after 3 weeks of lab-scale composting.

For BPE-RP-PLA, the resulting mass loss after 3 weeks of on-field industrial composting was much lower compared to the lab-scale equivalent. Although the reduction in mass is not significant on-field, BPE-RP-PLA had significant visual changes including yellowing and unevenness on the surface. Figure 4 depicts the on-field samples before and after industrial composting. In addition, the on-field samples became more brittle and prone to fragmentation. This signifies a structural degradation of the polymer matrix and indicates that the material was still within the incubation phase, theoretically representing a reduction in molecular weight before the start of mass loss due to structural disintegration.

For BPE-SP-PBS, the resulting mass loss after 3 weeks of on-field industrial composting was similar to the lab-scale equivalent, yet with a lower fragmentation of the samples. An average of 14% of the remaining mass after 3 weeks of composting in the lab was having a particle size between 2 mm and 10 mm, the fragmentation of the pieces > 10 mm was also much higher (see Figure 4 (e)). >99% for the on-field samples remained > 10 mm as most of the pieces remained intact except for small holes found in some pieces (see Figure 4 (d)). Data of the particle size distribution is given in *Underlying data* ( Chong *et al.*, 2022a). Additionally, BPE-SP-PBS on-field samples experienced yellowing and warping with noticeable holes. Though not captured by the remaining sample mass >2 mm measure, the samples in the second round lab-test are also further ahead in the disintegration process compared with the on-field test.

These observations indicate that rate of mass loss of biodegradable plastics will only be significant if the incubation phase is exceeded, as degradation of the material occurs at the beginning within the polymer matrix without causing structural disintegration. The extent of disintegration must also cross the size cutoff threshold of 2 mm before mass loss will be captured using the applied methodology. This indicates that even small differences in the duration of composting can result in large differences in remaining sample mass. The difference between the lab-scale and on-field test could be due to the differences in the compositing medium and conditions, which are further discussed in Differences in composting conditions between the lab-scale and on-field tests.

The results indicate that the tested blends in the current shape and form are not suitable for treatment in the industrial composting process where the test was carried out. Although degradation of the material was observed, the disintegration rate is very low within the active composting duration of the plant. It should be noted that the use of mesh bags might have negatively affected the disintegration rate by reducing the plastic-substrate contact area ( Sintim *et al.*, 2019). To minimize this risk, around 1 kg of organic waste (similar to the amount used in the lab-scale tests) was placed together with the samples in the mesh bags to facilitate direct contact. In addition, the thickness of the material play a deciding role in achieving sufficient disintegration. The suitability to treat compostable plastics in industrial composting plants in general is further discussed in Feasibility of treating compostable plastic packaging in industrial composting plants.

A parallel study using cellulose acetate-based films also showed low disintegration (<20%) after the industrial composting process ( Gadaleta *et al.*, 2023). In contrast, degradation rates up to 100% were found after 22 days of industrial composting of commercially available PLA based plant pot cuttings ( van der Zee & Molenveld, 2020). The study also reported a high variance of the disintegration rate between the PLA coffee capsule sample duplicates and attributed it to environmental variation. However, since exact dimensions of the samples were not given, a more detailed comparison is not possible. Another study reported that PLA/PBAT films only started to disintegrated after 21 days of exposure to industrial composting conditions ( Musioł *et al.*, 2018). It should be noted that comparison between on-field and pilot scale studies are difficult because of the different sample dimensions, substrate composition as well as general environmental conditions. In general, high variability can be seen from the outcomes of industrial composting.

### Differences in composting conditions between the lab-scale and on-field tests

The lab-scale tests based on ISO 20200 simulates a controlled thermophilic environment with a well-defined composting medium with small particle sizes (95% < 1 cm). On the other hand, the digestate material with which industrial composting was carried out had a larger and broader particle size distribution (shredded to ≤ 8cm). The composition would also change seasonally depending on separately collected organic waste from households. The measured pH, moisture content, volatile solids content and C/N ratio of the composting medium in both tests are listed in Table 2. The standard deviation of the total volatile solid measurements reflects the heterogeneity of the on-field composting medium, which had higher variation compared to the composting medium used in the lab.

**Table 2.** Lab-scale and on-field composting medium at the start of the experiment.

Test type	Lab-scale (3 weeks)	On-field
**Brief** **description**	A well-defined mix of materials based on ISO 20200.	A mixture of food and garden waste after dry anaerobic digestion. The composition depends on separately collected organic waste in the region.
**Particle size**	95% < 1cm	≤ 8cm
**Total dry solids ^1^**	45.0% (Start) ^4^ 29.0%±1.0% (End) ^5^	37.1%±5.0% (Start) 47.6%±1.9% (End)
**Total volatile** **solids ^2^**	90.6%±0.6%	37.8%±2.6%
**pH ^3^**	5.8±0.1	6.9±0.1
**C/N ratio ^3^**	33±2	19±1

1 Based on wet weight. 2 Based on total dry solids. 3 Measured on oven-dried and ground samples. 4 The moisture content was adjusted at the start of the experiment and thus not measured. 5 After three weeks of composting BPE-SP-PBS (Round 2).

The average volatile solid content of the lab-scale composting medium was higher compared to the on-field medium. Since the on-field medium stems from organic waste, it is expected that the inorganic content, i.e. sand and dust, will be higher due to the mixed collection of food and garden waste. The pH of the lab-scale composting medium was slightly acidic while the pH of the on-field medium was neutral; yet both in the accepted range for bacteria and fungi ( Chen *et al*., 2011). The C/N ratios of the lab-scale and on-field composting medium were close to the optimal for composting, quoted at 25 to 30 ( Kumar *et al*., 2010). Generally, it could be argued that the lab-scale composting medium with its high organic solids content, smaller particle sizes and thus homogeneity is more conducive to biotic disintegration as it facilitates a better sample-medium contact and higher biological activity. This could explain the better disintegration rates of the samples in the lab. In addition, the composting medium in the lab-scale test is mixed more frequently (based on the schedule in ISO 20200) compared to the on-field composting medium (once a week). The substrate particle size affects composting performance, where an optimal size after shredding increases the surface area to volume ratio, homogeneity, and also allows sufficient aeration and heating ( Amuah *et al.*, 2022; Reyes-Torres *et al.*, 2018). A higher particle size distribution and heterogeneity of the industrial composting medium might contribute to differing composting rates in different zones of the composting pile. In this study, the metal cages and mesh bag might have amplified the effects of heterogeneity by preventing the thorough mixing of the substrate within the cages with the rest of the substrate.

**Figure 5.**
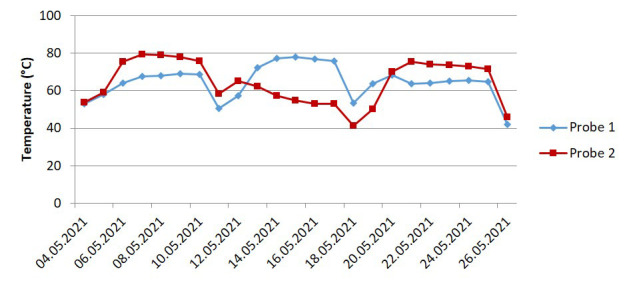
The temperature profile of the on-field industrial composting ( Bützberg Biogas and Composting Plant, 2021).

Factors affecting abiotic disintegration include temperature, pH and moisture. The temperature profiles of the on-field test differed slightly to the lab-scale tests. In the lab-scale experiments, the temperature was kept constant at 58°C via a convection oven. In contrast, the temperature of the on-field composting medium depended on self-heating and went through multiple cycles corresponding to the mixing schedules. The profiles of the temperature measured by the two probes are shown in Figure 5. The two dips in the middle correspond to the mixing phase, where the composting chambers are open and the composting medium is transferred from one chamber to another to facilitate mixing. The temperature ranged from 40°C to 80°C with an approximate average of 64°C. In both cases, the temperatures were well within the thermophilic range with the on-field composting having a higher average temperature.

Indicated by the measurements at the start and end of the experiments, the lab-scale tests environment had a lower moisture content compared to the on-field test at the start but a higher moisture content at the end. A wetter environment might facilitate better abiotic disintegration of PLA and PBS. The disintegration of PLA initially relies on chemical hydrolysis to break down long-chain polymers ( Mochizuki & Hirami, 1997) and thus benefits from higher humidity ( Karamanlioglu *et al*., 2017). The degradation of PBS relies on enzymatic activity on the surface ( Su *et al*., 2019) as well as hydrolysis ( Muthuraj *et al*., 2015) and thus would also benefit from increased moisture. Though, the slightly higher starting pH of the synthetic organic waste (pH 6) in comparison to the on-field substrate (pH 7) in the lab-scale could have positively contributed to the higher hydrolysis rates. On the other hand, a more basic pH was reported to accelerate surface erosion ( von Burkersroda *et al.*, 2002). The difference in terms of moisture content and pH is not a clear-cut in this study. Overall, the on-field test had better abiotic degradation conditions in terms of temperature. This did not however translate to higher disintegration compared to the lab-scale.

### Feasibility of treating compostable plastic packaging in industrial composting plants

Due to the complexity of the interaction between product design and composting parameters, the feasibility of treating compostable plastics in industrial composting plants depends on many factors in addition to the certification of the material. This study indicates that both blends in their current form do not meet the 90% disintegration requirement for compostable packaging according to EN 13432 after exposure to the process at the Bützberg plant. Although, BPE-SP-PBS did show sufficient disintegration in the lab-scale test after 12 weeks. A downside to the on-field tests is the use the sample nets and metal cages, which could have minimized the exposure to mechanical stress during the composting process, i.e. during mixing. In addition, the cages prevented thorough mixing of the substrate.

The disintegration of BPE-SP-PBS in other industrial composting plants might meet the requirement if the thermophilic composting phase is longer than 8 weeks, as indicated by the lab-scale test results. However, the thermophilic composting phase of most industrial processes does not exceed 3 weeks ( Ruggero *et al*., 2021). For BPE-RP-PLA, the thermophilic composting phase would need to be longer than 12 weeks to achieve sufficient degradation according to the lab-scale test results.

The conditions defined for certified compostable packaging, for example, those with their disintegration tested according to the pilot-scale disintegration test according to ISO 16929, are 4 weeks of composting above 55°C, 4 weeks of composting above 50°C and 4 weeks of composting below 45°C using fresh organic waste as inputs. These conditions also deviate significantly from those in Bützburg and similar plants with a shorter active composting phase. The German Ordinance on Biowastes requires hygienation temperatures of at least 55°C over two weeks and 60°C over 6 days during thermophilic composting ( BioAbfV, 2013). A sufficient degradation of the certified materials are thus not guaranteed solely based on these parameters.

Another important determining factor for disintegration is the shape and form of the biodegradable packaging articles, i.e. Tolga *et al*. (2020) found that the degree of disintegration linearly correlated with the thickness of PLA/PBS blends. Especially for rigid packaging, the thickness presents an extra obstacle as seen for BPE-RP-PLA. Extra pre-processing steps, i.e. shredding, would likely be needed to increase the disintegration of biodegradable plastics in industrial composting plants. Alternatively, thinner samples will theoretically allow for faster disintegration. However, research on the effects of differing substrate composition, particle size distribution and other parameters such as total volatile solids is lacking and important to understand the requirements for sufficient disintegration in actual plants as well as to understand the potential differences between plants.

It should be noted that the disintegration rate with a scope above 2 mm is not sufficient when considering the feasibility of treating compostable plastic in industrial composting plants. The behavior and ultimate biodegradation of the smaller compostable plastic particles in the environments in which they might enter, i.e. agricultural or garden soil, as well as their toxicity, should be determined ( Fojt *et al*., 2020; Karamanlioglu & Alkan, 2020; Sintim *et al.*, 2019). Lastly, integration with the wider waste management system needs to be considered. For one, the motivation of the industrial composting plants to sanitize organic waste and produce compost might not align with the treatment of compostable plastics, which might complicate the process without added benefit to the plant operator. In addition, the acceptance of biodegradable plastics in the organic waste stream increases the risk of contamination of conventional plastics, which are proven to be stable in industrial composting as well as anaerobic digestion ( Alassali *et al.*, 2018).

## Conclusion

Heeding the resistance from some waste management authorities to treat compostable plastics in composting plants, the behavior of biodegradable and compostable plastics under actual industrial composting conditions should be thoroughly examined to assess their environmental impact. Therefore, this study investigated the disintegration rate of a PLA blend and a PBS blend designed for rigid and soft packaging respectively under simulated lab-scale and on-site industrial composting conditions.

While under simulated industrial conditions according to ISO 20200, BPE-RP-PLA and BPE-SP-PBS samples achieved significant disintegration with averages of 55.7% and 0.3% respectively after 12 weeks, showing good disintegration potential. However, the mass loss was low after on-field composting with remaining masses of at least 97.2% for the BPE-RP-PLA samples and 93.8% for the BPE-SP-PBS samples after 3 weeks. This stark difference is not solely due to the shorter composting duration on-field, as the supplementary lab-scale test of the same duration showed higher mass loss for BPE-RP-PLA with an average of 68.9% remaining mass. BPE-SP-PBS achieved similar mass loss after 3 weeks in the lab but showed more fragmentation with 14% of its remaining mass under 10 mm particle size compared to <1% for the on-field samples. Based on the data in this study, the abiotic conditions such temperature, moisture content and pH in the lab-scale was not notably better compared to on-field. The methods used also ensured that at least 1 kg of composting substrate was in direct contact with the plastic samples in all cases. This point towards the potential role of other composting parameters such as the process scale, substrate composition and particle size distribution in the rate of disintegration. These factors may vary for different plants and seasonally. In addition, the presence and diversity of microorganisms play a role. More research is needed in order to understand their effects on biodegradable plastic products.

Although BPE-SP-PBS samples in their current thickness almost disintegrated entirely after 12 weeks, a minimum thermophilic composting duration of 8 weeks would be necessary to reach the 90% disintegration (10% remaining mass) required by EN 13432, which exceeds the thermophilic phase of most composting plants. Biological waste treatment plants including composting plants are generally designed to treat and sanitize biological waste and are not optimized to treat compostable plastics, especially concerning the retention time. Henceforth, realistic certification requirements that reflect differing retention times and substrate composition which occur in real-life applications should be considered. Furthermore, systemic integration considering the organic waste value chain and the motivations of industrial composting plants is critical.

## Data Availability

B2Share: Underlying data_Lab-scale and on-field industrial composting of biodegradable plastic blends for packaging_Chong *et al*., 2023 https://doi.org/10.23728/b2share.f1e07485392443cfa455c9745f4d95ec ( Chong *et al*., 2022a) This project contains the following underlying data: CN ratio_Industrial test.xlsx CN ratio_lab-scale.xlsx Data list.docx Moisture content.xlsx Particle size distribution after composting_BPE-SP-PBS.xlsx pH.xlsx Remaining mass industrial test.xlsx Remaining mass lab-scale_BPE-RP-PLA.xlsx Remaining mass lab-scale_BPE-SP-PBS.xlsx R value lab-scale test reactors_BPE-RP-PLA.xlsx R value lab-scale test reactors_BPE-SP-PBS.xlsx Total volatile solids.xlsx B2Share: Extended data_Lab-scale and on-field industrial composting of biodegradable plastic blends for packaging_Chong *et al*., 2022 http://doi.org/10.23728/b2share.5356b112d6bd424aa79b10d397ee1b81 ( Chong *et al*., 2022b) This project contains the following extended data: Extended data_Methodology.docx Data are available under the terms of the Creative Commons Attribution 4.0 International license (CC-BY 4.0).
