# Supplementary material for: Lab-scale and full-scale industrial composting of biodegradable plastic blends for packaging
Source: Open Res Eur. 2024 Jan 22;2:101. Originally published 2022 Aug 23. [Version 3] doi: 10.12688/openreseurope.14893.3 (PMC10899788; doi:10.12688/openreseurope.14893.3)
Supplement: Peer review history

# Reviewer report: Reviewer response for version 3

**DOI:** 10.21956/openreseurope.18403.r37653

**Published:** 2024-02-27

**Authors:** Mehlika Karamanlioglu

**Affiliations:** 1 Faculty of Engineering and Architecture, Department of Biomedical Engineering, Istanbul Gelisim University, İstanbul, Turkey

I have no further comments since the responses are satisfactory.

Is the study design appropriate and does the work have academic merit?

Yes

Is the work clearly and accurately presented and does it cite the current literature?

Partly

If applicable, is the statistical analysis and its interpretation appropriate?

Not applicable

Are all the source data underlying the results available to ensure full reproducibility?

Yes

Are the conclusions drawn adequately supported by the results?

Partly

Are sufficient details of methods and analysis provided to allow replication by others?

Yes

Reviewer Expertise:

Biotechnology, biopolymers, environmental degradation of plastics.

I confirm that I have read this submission and believe that I have an appropriate level of expertise to confirm that it is of an acceptable scientific standard.

# Reviewer report: Reviewer response for version 3

**DOI:** 10.21956/openreseurope.18403.r37652

**Published:** 2024-02-12

**Authors:** Douglas G. Hayes

**Affiliations:** 1 Department of Biosystems Engineering & Soil Science, University of Tennessee, Knoxville, TN, USA

The authors have addressed all of the issues I raised satisfactorily. I therefore recommend that this manuscript be accepted for indexing.

Is the study design appropriate and does the work have academic merit?

Yes

Is the work clearly and accurately presented and does it cite the current literature?

Yes

If applicable, is the statistical analysis and its interpretation appropriate?

Partly

Are all the source data underlying the results available to ensure full reproducibility?

Yes

Are the conclusions drawn adequately supported by the results?

Partly

Are sufficient details of methods and analysis provided to allow replication by others?

Yes

Reviewer Expertise:

Biobased and biodegradable agricultural plastics, micro- and nanoplastics, colloidal systems

I confirm that I have read this submission and believe that I have an appropriate level of expertise to confirm that it is of an acceptable scientific standard.

# Reviewer report: Reviewer response for version 2

**DOI:** 10.21956/openreseurope.17887.r35107

**Published:** 2023-10-20

**Authors:** Mehlika Karamanlioglu

**Affiliations:** 1 Faculty of Engineering and Architecture, Department of Biomedical Engineering, Istanbul Gelisim University, İstanbul, Turkey

There are some significant improvements in the revised paper. However, there are still some parts that need more explanation. Further comments are made below:

 1 - The main aim of this study should be clearly presented especially in the background and in the introduction. What do you aim to compare? The degradation rate of 2 plastics, or only the degradation rates in the lab scale and in the composting site? If it is the latter one, the comparison on the 3 ^rd^ week can enable to achieve this aim.

 Therefore, why did you conduct the tests on the 4 ^th^, 8 ^th^ and 12 ^th^ week in lab scale?  It should be written and explained clearly in relevant sections.

 2 - The term 'on-field' is confusing. Industrial composting vs. lab scale sounds more accurate.

 3 - The supplementary lab-scale test for 3 weeks should be written in lab-scale industrial composting part in the method with more detail. This is actually an important test so it should be emphasized more in the methods part rather than being just a supplementary test. 

 Also  it is written for the supplementary test that 'This test was done in triplicate'. Could you elaborate on that? 

 4 - Burial of samples in lab scale test should be given in more detail. Were they directly buried in the pile?

 5 - Although molecular weight and Tg are given, still more detail about the plastics used in this study should be given. What does BPE stand for, how did you obtain these plastics, did you make them or buy them commercially, i.e., were they commercial packages such as deli containers, etc.? It is mentioned that ‘…two biodegradable plastic prototypes developed for rigid (PLA-based) and soft packaging (PBS-based) respectively within the BIO-PLASTICS EUROPE research Project…’ How did you develop them? It should be added to methods parts as materials used.

 6 - In the industrial composting test, why do you have 2 results for the same plastic (Field sample 1 and 2)? It is written that:  ‘In the plant, the remaining mass was 97.2% and 99.5%’, Do these plastics have any difference? If there is no difference, if they are only duplicates, you should present the average of them with standard deviation or standard error of the mean.  

 7 - Cite the source after these sentences: ‘The authors attributed this to a higher autocatalytic degradation phenomenon in PLA. The same study also showed that thickness has a strong effect on the disintegration speed.’

Is the study design appropriate and does the work have academic merit?

Yes

Is the work clearly and accurately presented and does it cite the current literature?

Partly

If applicable, is the statistical analysis and its interpretation appropriate?

Not applicable

Are all the source data underlying the results available to ensure full reproducibility?

Yes

Are the conclusions drawn adequately supported by the results?

Partly

Are sufficient details of methods and analysis provided to allow replication by others?

Yes

Reviewer Expertise:

Biotechnology, biopolymers, environmental degradation of plastics.

I confirm that I have read this submission and believe that I have an appropriate level of expertise to confirm that it is of an acceptable scientific standard, however I have significant reservations, as outlined above.

# Response

**Published:** 2024-01-22

**Authors:** Zhi Kai Chong

**Affiliations:** Hamburg University of Technology, Germany

*The authors would like to thank Mehlika Karamanlioglu for the helpful review and comments to improve the structure.* *The edits made in response to each comment as well as explanations are given point-by-point below in italics.*

 1 - The main aim of this study should be clearly presented especially in the background and in the introduction. What do you aim to compare? The degradation rate of 2 plastics, or only the degradation rates in the lab scale and in the composting site? If it is the latter one, the comparison on the 3 ^rd^ week can enable to achieve this aim.

 Therefore, why did you conduct the tests on the 4 ^th^, 8 ^th^ and 12 ^th^ week in lab scale?  It should be written and explained clearly in relevant sections. *Thank you for the comment. To improve the description of the study’s aim, the last paragraph of the introduction was revised to “The controlled, lab-scale test represented the disintegration potential of the prototypes and was conducted for a duration up to 12 weeks, the maximum allowed composting duration based on EN 13432. On the other hand, the test in the composting plant represented a real-life scenario. The composting medium of both cases was characterized for comparison. The results outlined the difference of disintegration rates in the lab and in a plant and explored the possible reasons for the differences.”.* *The main aim of the study was to investigate the behavior of compostable plastic packaging prototypes in real-life composting conditions as well as the relation to their behavior in a controlled, lab-scale test.* *The composting test in the lab was necessary for up to 12 weeks to determine the disintegration potential of the material (ISO 20200 and EN 13432 allows for composting up to 12 weeks). In addition, this allows us to make inferences on the behavior of the material in other composting plants that might have a longer thermophilic composting duration compared to the one presented in this study.*

 2 - The term 'on-field' is confusing. Industrial composting vs. lab scale sounds more accurate. *“On-field” was replaced by “full-scale”. The description “full-scale” is still needed because both “lab-scale” and “full-scale” tests refer to industrial composting conditions, which is characterized as thermophilic composting.*

 3 - The supplementary lab-scale test for 3 weeks should be written in lab-scale industrial composting part in the method with more detail. This is actually an important test so it should be emphasized more in the methods part rather than being just a supplementary test.

 Also  it is written for the supplementary test that 'This test was done in triplicate'. Could you elaborate on that? *The text mentioned was moved to the Section “Lab-scale industrial composting” to facilitate easier understanding. The text was also revised to make it clear that it is relevant for comparison between the lab-scale and full-scale composting conditions (temperature and substrate mix).* *“Triplicate” refered to three separate composting instances (boxes) for each time point. The results were used to calculate the average and standard deviation for each time point.*

 4 - Burial of samples in lab scale test should be given in more detail. Were they directly buried in the pile? *We ensured that >80% of the samples were covered by the substrate in the lab-scale test. Due to the high surface area to mass ratio, a 100*% coverage was not *possible. Further explanations were added as suggested to paragraph 3 of the Section “Lab-scale industrial composting”.*

 5 - Although molecular weight and Tg are given, still more detail about the plastics used in this study should be given. What does BPE stand for, how did you obtain these plastics, did you make them or buy them commercially, i.e., were they commercial packages such as deli containers, etc.? It is mentioned that ‘…two biodegradable plastic prototypes developed for rigid (PLA-based) and soft packaging (PBS-based) respectively within the BIO-PLASTICS EUROPE research Project…’ How did you develop them? It should be added to methods parts as materials used. *The last paragraph of the section “Biodegradable plastic test samples” was revised to make the source of the samples clearer.* *The plastic prototypes were not obtained from commercial sources but produced by other partners within the BIO-PLASTICS EUROPE project. Though the formulations (blends) were developed within the project, the formulations and their effects on disintegration was not part of this study. This study focused on the potential disintegration behaviour of compostable plastic packaging products in industrial composting conditions.*

 6 - In the industrial composting test, why do you have 2 results for the same plastic (Field sample 1 and 2)? It is written that:  ‘In the plant, the remaining mass was 97.2% and 99.5%’, Do these plastics have any difference? If there is no difference, if they are only duplicates, you should present the average of them with standard deviation or standard error of the mean. *Thank you for the suggestion, we have updated the figure as suggested. The full-scale test data was presented as a mean with standard deviation. The text were also updated to reflect the figures presented.*

 7 - Cite the source after these sentences: ‘The authors attributed this to a higher autocatalytic degradation phenomenon in PLA. The same study also showed that thickness has a strong effect on the disintegration speed.’` *The text was revised to make it clear that these sentences are also cited from the study cited in the previous sentence “Comparing PLA and PBS, PLA/PBS blends with a higher ratio of PLA were reported to achieve higher disintegration rates in industrial composting conditions by Tolga et al. (* *Tolga *et al. *, 2020* *).”*

# Reviewer report: Reviewer response for version 2

**DOI:** 10.21956/openreseurope.17887.r35106

**Published:** 2023-10-11

**Authors:** Douglas G. Hayes

**Affiliations:** 1 Department of Biosystems Engineering & Soil Science, University of Tennessee, Knoxville, TN, USA

Assessment of revised version of “Lab-scale and on-field industrial composting of biodegradable plastic blends for packaging”, Chong ZK, Hofmann A, Haye M, Wilson S, Sohoo I, Alassali A and Kuchta K

 Assessor: Douglas G. Hayes, Biosystems Engineering, University of Tennessee (USA)

 I find the revised version of the manuscript is greatly improved. I recommend acceptance after the following minor revisions are addressed.

1. Why is the term “on-field” used for the industrial composting test? Industrial composting took place in a composting facility in Tangstedt, Germany, not in an agricultural field. I find this term to be confusing and perhaps misleading, and recommend that it be replaced with “industrial composting” (or equivalent) throughout the manuscript, including figures and tables.
2. Introduction, paragr 1, “development of alternatives” to what? Unclear.
3. Introduction, paragr 1, “decomposition of carbon” is awkward, unless a nuclear fission reaction is being referred to.
4. Introduction, paragr 1, “Compostable plastics .. that disintegrate significantly”. This statement is of concern to me, since oxodegradables can “disintegrate significantly” but will not disappear. If the term “compostable” is employed with regard to a standard such as ISO 14855, the plastic material needs to fulfill several criteria pertaining not only to disintegration, but also biodegradation and heavy metal content. This sentence should be revised.
5. Introduction, paragr 2, “Industrially compostable products are designed to disintegrate ..” For the same rationale as provided above for Remark 4, this sentence needs to be revised. Oxodegradables are designed to “disintegrate” but are not compostable because of their inability to fully biodegrade.
6. Introduction, paragr 4, “has been studied in the literature in the lab ..” is awkward. The sentence would suggest that the scientists have conducted their literature searches in their lab rather than their office, or elsewhere.
7. Introduction, paragr 4: Please provide units for the ratio “7:3”, such as “w/w”, “mol/mol”, or “v/v”.
8. Introduction, paragr 7, “Chemically, the disintegration of PLA .. microbiological enzymatic attack”. Are abiotic reactions limited to hydrolysis? Can PLA undergo photodegradative reactions in the presence of sunlight that differ from hydrolysis?
9. For Table 1, why are there error bars for only some, but not all, of the entries?
10. On-field industrial composting test” section, paragr 1. Please describe greater detail on the “magnetic separation” procedure used to prepare the compost. Can a reference be cited?
11. Results and Discussion section, paragr 3; Grammar error (verb tense): “Comparing PLA and PBS ..blends with a higher ratio .. was reported”.
12. “Disintegration rate in an industrial composting plant” subsection, paragr 3: “Though not captured by the remaining ..”: “mass > 2 mm measure” is awkward. Also, what is meant by “second round lab-test”? I do not recall seeing anything related in the Experimental section.
13. “Disintegration rate in an industrial composting plant” subsection, paragr 4, “significant if the incubation phase is exceeded” : “exceeded” is awkward / unclear.
14. “Disintegration rate in an industrial composting plant” subsection, paragr 6, “only started to disintegrated” (grammar).
15. “Differences in composting conditions between ..” subsection, first paragr: Please insert a space between “8” and “cm”. Please also insert spaces between the numerical values and the units in Table 2, “particle size’ row.
16. For Table 2, I see that the numbering scheme for the footnotes is down the columns moving left to right. For many journals, the preference is to number from left to right across a column, and ten move down the rows. Please check with the journal on the preferred format.
17. “Differences in composting conditions between ..” subsection,, paragr 2: “optimal size after shredding increases the surface area to volume ratio”. Actually, the trend of increasing SA/V with decreasing size occurs both above and below an optimal size. Please reword this sentence.
18. “Differences in composting conditions between ..” subsection, paragr 3: “dips” is slang. Suggest replacing with “minima”.
19. “Differences in composting conditions between ..” subsection, paragr 4: “The degradation of PBS relies on enzymatic activity .. as well as hydrolysis” Since hydrolysis can be abiotic or biotic, this sentence is a little awkward. I suggest using “as well as abiotic hydrolysis” to clarify.
20. Paragraph above Conclusions: “scope” is a little awkward. (“..the disintegration rate with a scope above 2 mm..”)

Is the study design appropriate and does the work have academic merit?

Yes

Is the work clearly and accurately presented and does it cite the current literature?

Yes

If applicable, is the statistical analysis and its interpretation appropriate?

Partly

Are all the source data underlying the results available to ensure full reproducibility?

Yes

Are the conclusions drawn adequately supported by the results?

Partly

Are sufficient details of methods and analysis provided to allow replication by others?

Yes

Reviewer Expertise:

Biobased and biodegradable agricultural plastics, micro- and nanoplastics, colloidal systems

I confirm that I have read this submission and believe that I have an appropriate level of expertise to confirm that it is of an acceptable scientific standard, however I have significant reservations, as outlined above.

# Response

**Published:** 2024-01-22

**Authors:** Zhi Kai Chong

**Affiliations:** Hamburg University of Technology, Germany

*The authors would like to thank Douglas G. Hayes for the review. We appreciate the comments and the attention to detail. The edits made in response to each comment as well as explanation are given point-by-point below in italics.*

1. Why is the term “on-field” used for the industrial composting test? Industrial composting took place in a composting facility in Tangstedt, Germany, not in an agricultural field. I find this term to be confusing and perhaps misleading, and recommend that it be replaced with “industrial composting” (or equivalent) throughout the manuscript, including figures and tables.

*“On-field” was replaced by “full-scale”. The description “full-scale” is still needed because both “lab-scale” and “full-scale” tests refer to industrial composting conditions, which is characterized as thermophilic composting.*

1. Introduction, paragr 1, “development of alternatives” to what? Unclear.

*The sentence was revised to “The prevalence of plastic waste and micro-plastic in the environment has spurred research and development of biodegradable plastics as alternatives to conventional fossil-based non-biodegradable plastics.”.*

1. Introduction, paragr 1, “decomposition of carbon” is awkward, unless a nuclear fission reaction is being referred to.

*The sentence was revised to “It is important to note the difference between the terms biodegradation and disintegration. In EN 13432, biodegradability is to be proved via the decomposition of the polymer structure and the subsequent release of the carbon in the plastic material ultimately as carbon dioxide.”*

1. Introduction, paragr 1, “Compostable plastics .. that disintegrate significantly”. This statement is of concern to me, since oxodegradables can “disintegrate significantly” but will not disappear. If the term “compostable” is employed with regard to a standard such as ISO 14855, the plastic material needs to fulfill several criteria pertaining not only to disintegration, but also biodegradation and heavy metal content. This sentence should be revised.

*We appreciate the comment. We regard the disintegration ability as a property of plastics that are inherently compostable without referring to a specific standard. The heavy metal content would be part of a certification and/or standard. We thus differentiate here between (generally) compostable plastics and certified compostable plastics, the latter which follows a certain certification. In this context, we are referring to the former and thus prefer to keep the sentence as it is. The ultimate biodegradability is implied with the use of the term “biodegradable plastics”.*

1. Introduction, paragr 2, “Industrially compostable products are designed to disintegrate ..” For the same rationale as provided above for Remark 4, this sentence needs to be revised. Oxodegradables are designed to “disintegrate” but are not compostable because of their inability to fully biodegrade.

*The sentence was revised to “Industrial compostable products are produced from biodegradable material and designed to disintegrate within a reasonable period under industrial composting conditions.”*

1. Introduction, paragr 4, “has been studied in the literature in the lab ..” is awkward. The sentence would suggest that the scientists have conducted their literature searches in their lab rather than their office, or elsewhere.

*The sentence was revised to “In literature, the behavior of biodegradable plastics in the composting process has been studied in both lab- and pilot scales.”*

1. Introduction, paragr 4: Please provide units for the ratio “7:3”, such as “w/w”, “mol/mol”, or “v/v”.

*“w/w” was added to the sentence.*

1. Introduction, paragr 7, “Chemically, the disintegration of PLA .. microbiological enzymatic attack”. Are abiotic reactions limited to hydrolysis? Can PLA undergo photodegradative reactions in the presence of sunlight that differ from hydrolysis?

*We agree with the comment, PLA may also degrade via other abiotic pathways such as thermal-degradation and photodegradation. However, biodegradable polyesters were reported to be particularly susceptible to hydrolysis (Woodard und Grunlan 2018; Speranza et al. 2014) and thus can be regarded as the main abiotic degradation mechanism in industrial composting conditions. With the absence of sunlight, the photodegradative pathway should be less relevant.*

1. For Table 1, why are there error bars for only some, but not all, of the entries?

*That is a good question. We added the standard deviation for the film thickness because it has significant variability due to the fact that it was a prototype produced in the lab. We added an explanation for the standard deviation in the captions.*

1. On-field industrial composting test” section, paragr 1. Please describe greater detail on the “magnetic separation” procedure used to prepare the compost. Can a reference be cited?

*The sentence was revised to: “The plant receives separately collected bio-waste from households in the region. Before undergoing biological treatment, the waste was shredded (< 8 cm), sieved and sent through magnetic separation to remove impurities such as plastics and metals.”. The magnetic separation process is carried out on the organic waste inputs before composing. We received this information through personal communication with the industrial plant and thus can not provide a reference. However, impressions of the plant can be seen on this website: https://www.stadtreinigung.hamburg/ueber-uns/muellverwertung-anlagen/biogas-und-kompostwerk-buetzberg/*

1. Results and Discussion section, paragr 3; Grammar error (verb tense): “Comparing PLA and PBS ..blends with a higher ratio .. was reported”.

*The sentence was corrected.*

1. “Disintegration rate in an industrial composting plant” subsection, paragr 3: “Though not captured by the remaining ..”: “mass > 2 mm measure” is awkward. Also, what is meant by “second round lab-test”? I do not recall seeing anything related in the Experimental section.

*The sentence was revised to: “Additionally, BPE-SP-PBS full-scale samples experienced yellowing and warping with noticeable holes and thus were also in a more advance stage of the disintegration process compared with the full-scale test in this aspect.” to reduce confusion.*

1. “Disintegration rate in an industrial composting plant” subsection, paragr 4, “significant if the incubation phase is exceeded” : “exceeded” is awkward / unclear.

*The sentence was revised to: “These observations indicate that the rate of mass loss of biodegradable plastics will only be significant if the duration of the thermophilic composting phase exceeds the incubation phase, as degradation of the material occurs at the beginning within the polymer matrix without causing structural disintegration.”.*

1. “Disintegration rate in an industrial composting plant” subsection, paragr 6, “only started to disintegrated” (grammar).

*The sentence was corrected.*

1. “Differences in composting conditions between ..” subsection, first paragr: Please insert a space between “8” and “cm”. Please also insert spaces between the numerical values and the units in Table 2, “particle size’ row.

*The text was corrected.*

1. For Table 2, I see that the numbering scheme for the footnotes is down the columns moving left to right. For many journals, the preference is to number from left to right across a column, and ten move down the rows. Please check with the journal on the preferred format.

*We checked this with the editors and made the necessary corrections.*

1. “Differences in composting conditions between ..” subsection,, paragr 2: “optimal size after shredding increases the surface area to volume ratio”. Actually, the trend of increasing SA/V with decreasing size occurs both above and below an optimal size. Please reword this sentence.

*The sentence was revised to: “The substrate particle size affects composting performance, shredding increases the surface area to volume ratio, homogeneity, and also allows sufficient aeration and heating as long as the resulting substrate size is within an optimal range (* *Amuah *et al. *, 2022* *;* *Reyes-Torres *et al. *, 2018* *).”*

1. “Differences in composting conditions between ..” subsection, paragr 3: “dips” is slang. Suggest replacing with “minima”.

*The text was corrected.*

1. “Differences in composting conditions between ..” subsection, paragr 4: “The degradation of PBS relies on enzymatic activity .. as well as hydrolysis” Since hydrolysis can be abiotic or biotic, this sentence is a little awkward. I suggest using “as well as abiotic hydrolysis” to clarify.

*“abiotic” was added to the sentence.*

1. Paragraph above Conclusions: “scope” is a little awkward. (“..the disintegration rate with a scope above 2 mm..”)

*The sentence was revised to: “It should be noted that the disintegration rate calculated considering a size cutoff of 2 mm is not sufficient when considering the feasibility of treating compostable plastic in industrial composting plants.”*     Literature Speranza, V.; Meo, A. de; Pantani, R. (2014): Thermal and hydrolytic degradation kinetics of PLA in the molten state. In: *Polymer Degradation and Stability *100, S. 37–41. DOI: 10.1016/j.polymdegradstab.2013.12.031. Woodard, Lindsay N.; Grunlan, Melissa A. (2018): Hydrolytic Degradation and Erosion of Polyester Biomaterials. In: *ACS macro letters *7 (8), S. 976–982. DOI: 10.1021/acsmacrolett.8b00424.

# Reviewer report: Reviewer response for version 1

**DOI:** 10.21956/openreseurope.16095.r30286

**Published:** 2022-11-01

**Authors:** Douglas G. Hayes

**Affiliations:** 1 Department of Biosystems Engineering & Soil Science, University of Tennessee, Knoxville, TN, USA

I find this manuscript to be publishable after addressing minor revisions. The experimental work is reliable and was performed correctly, per ISO 16929 and 20200 standardized testing conditions for measuring disintegration. The interpretation of the data is mostly correct, in my opinion. However, there are a few places where I believe interpretation should be reconsidered. First, one must be careful on extending results achieved using standardized tests and specifications achieved per a standard to actual performance in an industrial composting facility. As the authors likely know, composting is truly an art and its performance can be affected by several different parameters. Second, biodegradation is directly controlled by the surface area per volume of the plastic material. This is relevant in two contexts: A) The PLA test material has a 10-fold higher thickness compared to PBS, which will lead to a lower biodegradation rate for PLA, and B) The mesh bags used in the study will likely introduce a resistance to mass transfer of nutrients and gases across the mesh bag matrix. In addition, the presentation can be improved in a few places in the manuscript.

 Regarding the mass transfer inhibition of biodegradation (disintegration) caused by the mesh bag, I would recommend performing a side-by-side laboratory test of a standard plastic testing material (e.g., a thin PLA film) directly in compost vs in a mesh bag placed in compost. Such an experiment does not need to be carried out for the full duration of the experiments described in your paper; perhaps a few weeks would suffice. We did this: H.Y. Sintim, et al., 2019 ^1^ (within Supplementary Materials) and found for our systems that the mesh bags significantly slowed the biodegradation rate in compost.

 My specific comments are given below.

1. Introduction, first paragraph: I would recommend defining “disintegration” and “composting” carefully. EN 13432 or one of the ISO disintegration testing methods should have these definitions. I disagree with to the use of “only” (“..would ONLY disintegrate significantly..”). A substance’s ability to readily biodegrade under composting conditions does not preclude the substance’s ability to readily biodegrade in a different environment (e.g., soil, activated sludge, or seawater).
2. Introduction, 3 ^rd^ paragraph: “The disintegration condition in EN 13432 .. after a maximum of 12 weeks”. This sentence needs to be qualified: “..according to the _______ standardized testing conditions”
3. More information should be provided on the properties of the polymeric materials, such as weight-averaged molecular weight, glass transition temperature, apparent density (grams per square meter), etc.
4. Methods, Lab-scale composting. Were the composting ingredients or the compost itself treated with sieving prior to the addition of plastic?
5. Results and Discussion, first paragraph, “current form does not fulfil the 90% disintegration conditions” needs a qualifier, such as “specified in EN 13432”
6. Please consider merging Figs 2 and 3. The data of Fig 3 can be easily incorporated as datapoints into Fig 2. (Note, for the current Fig 3, y-axis numbers should have the zeroes to the right of the decimal place removed and the caption should indicate the duration.)
7. Fig 2: Do error bars represent standard deviation, standard error, or something else? Please elaborate (e.g., in the figure caption).
8. Results and discussion, “disintegration rate in an industrial composting plant” subsection, bottommost paragraph. This paragraph needs revision. First, as mentioned above, it is likely that the mesh bag promoted mass transfer limitations on the biodegradation reaction. Second, I believe that the non-compliance of PLA to the EN 13432 standards is at least partially attributable to the high, 1 mm, thickness of the films and perhaps the high content of calcium silicate nanofiller may play a role as inhibitor of biodegradation. Are the composting conditions and medium a factor? I find that the inclusion of a positive control into the experimental design, such as a standard plastic material (e.g., a thinner PLA film) improves in the interpretation of the results, although I realize that the ISO standardized tests for disintegration do not directly call for it. Please consider revising the discussion in this paragraph.
9. Feasibility of treating compostable plastic packaging .. “ subsection, first paragraph, “do not meet the 90% disintegration requirement”. I find this paragraph requires revision, noting first that the PBS material does appear to meet the requirement under laboratory conditions, per Fig 2. The assessment given in this paragraph needs to take into account the high thickness of the PLA film and the mass transfer limitations imposed by the mesh bag for the data of Fig 3 (as discussed above)

Is the study design appropriate and does the work have academic merit?

Yes

Is the work clearly and accurately presented and does it cite the current literature?

Yes

If applicable, is the statistical analysis and its interpretation appropriate?

Partly

Are all the source data underlying the results available to ensure full reproducibility?

Yes

Are the conclusions drawn adequately supported by the results?

Partly

Are sufficient details of methods and analysis provided to allow replication by others?

Yes

Reviewer Expertise:

Biobased and biodegradable agricultural plastics, micro- and nanoplastics, colloidal systems

I confirm that I have read this submission and believe that I have an appropriate level of expertise to confirm that it is of an acceptable scientific standard, however I have significant reservations, as outlined above.

## References

[1] : Release of micro- and nanoparticles from biodegradable plastic during in situ composting. *Sci Total Environ* .2019;675: 10.1016/j.scitotenv.2019.04.179 686-693 10.1016/j.scitotenv.2019.04.179 31039503

# Response

**Published:** 2023-08-25

**Authors:** Zhi Kai Chong

**Affiliations:** Hamburg University of Technology, Germany

The authors would like to thank Douglas G. Hayes for the detailed review and constructive comments. The table below summarises the authors’ comments and enhancements made to the new version in response to the review. Review Section Authors’ replies and comments I find this manuscript to be publishable after addressing minor revisions. The experimental work is reliable and was performed correctly, per ISO 16929 and 20200 standardized testing conditions for measuring disintegration. The interpretation of the data is mostly correct, in my opinion. However, there are a few places where I believe interpretation should be reconsidered. First, one must be careful on extending results achieved using standardized tests and specifications achieved per a standard to actual performance in an industrial composting facility. As the authors likely know, composting is truly an art and its performance can be affected by several different parameters. Second, biodegradation is directly controlled by the surface area per volume of the plastic material. This is relevant in two contexts: A) The PLA test material has a 10-fold higher thickness compared to PBS, which will lead to a lower biodegradation rate for PLA, and B) The mesh bags used in the study will likely introduce a resistance to mass transfer of nutrients and gases across the mesh bag matrix. In addition, the presentation can be improved in a few places in the manuscript. The authors agree that extending results from standardized test to industrial composting plant is highly problematic. The difference between our results in the lab compared to the industrial composting plant is evidence of that. The possible reasons for the difference in disintegration rates were enhanced in the section “Differences in composting conditions between the lab-scale and on-field tests”, paragraph 2 to 4.   The specific surface area of the samples were added in Table 1. In addition, the influence of the specific surface area on the results comparing the film and plate samples were added in the Section “Disintegration rate in lab-scale industrial composting”,paragraph 2 and 3.   A discussion of the potential effect of the mesh bags was also included in the section “Disintegration rate in an industrial composting plant”,paragraph 5. Regarding the mass transfer inhibition of biodegradation (disintegration) caused by the mesh bag, I would recommend performing a side-by-side laboratory test of a standard plastic testing material (e.g., a thin PLA film) directly in compost vs in a mesh bag placed in compost. Such an experiment does not need to be carried out for the full duration of the experiments described in your paper; perhaps a few weeks would suffice. We did this: H.Y. Sintim, et al., 20191 (within Supplementary Materials) and found for our systems that the mesh bags significantly slowed the biodegradation rate in compost. Thank you for the helpful comments. A discussion of the potential effect of the mesh bags was added to the section “Disintegration rate in an industrial composting plant”,paragraph 5. In addition, the potential effect of using mesh bags coupled with the heterogeneity of the substrate is mentioned in the section “Differences in composting conditions between the lab-scale and on-field tests”,paragraph 2. Due to the presence of 1 kg of organic waste directly in contact with the samples in the on-field test, we believe the risk of reduced contact is minimal. However, the substrate within the mesh bags might have lower disintegration rates considering reduced mixing with the rest of the compost pile. 1.            Introduction, first paragraph: I would recommend defining “disintegration” and “composting” carefully. EN 13432 or one of the ISO disintegration testing methods should have these definitions. I disagree with to the use of “only” (“..would ONLY disintegrate significantly..”). A substance’s ability to readily biodegrade under composting conditions does not preclude the substance’s ability to readily biodegrade in a different environment (e.g., soil, activated sludge, or seawater). Definitions for biodegradation and disintegration were added in section “Introduction”, paragraph 1. In addition, a sentence rephrased to “Compostable plastics are a subcategory of biodegradable plastics that disintegrate significantly in specific composting conditions, but not necessarily in general environmental conditions on land or sea.” 2.            Introduction, 3rd paragraph: “The disintegration condition in EN 13432 .. after a maximum of 12 weeks”. This sentence needs to be qualified: “..according to the _______ standardized testing conditions” Qualifier added “in a “controlled pilot-scale test” or in industrial composting plants” 3.            More information should be provided on the properties of the polymeric materials, such as weight-averaged molecular weight, glass transition temperature, apparent density (grams per square meter), etc. The weight average molecular weight, glass transition temperature and specific surface area  (square centimeter per gram) were added to Table 1. Due to the lack of data, the average molecular weight of BPE-SP-PBS is not available. The glass transition temperature typical for PBS was extracted from literature. The discussion in paragraph 2 and 3 of the section “Disintegration rate in lab-scale industrial composting” was also enhanced using the added information. 4.            Methods, Lab-scale composting. Were the composting ingredients or the compost itself treated with sieving prior to the addition of plastic? The substrate mix was not treated with sieving prior to the test. This is because the components were based on ISO 20200 and were sourced from homogeneous sources. From our sieving analysis, at least 95% of the substrate had a particle size less than 1 cm. The compost itself was sieved in the production line of the composting plant. On visual inspection, we did not identify plastic particles that may affect the results of the experiment. 5.            Results and Discussion, first paragraph, “current form does not fulfil the 90% disintegration conditions” needs a qualifier, such as “specified in EN 13432” Qualifier added “based on ISO 20200”. 6.            Please consider merging Figs 2 and 3. The data of Fig 3 can be easily incorporated as datapoints into Fig 2. (Note, for the current Fig 3, y-axis numbers should have the zeroes to the right of the decimal place removed and the caption should indicate the duration.) That is a good point, thank you. We added additional data for a lab-scale test for 3 weeks to Figure 3 reflecting the duration as well as material dimensions used in the on-field test in the industrial composting plant. We also edited the figure as suggested. Due to the differing dimensions of the samples used for the 1 ^st^ lab-scale test, we prefer to keep the figures separate. 7.            Fig 2: Do error bars represent standard deviation, standard error, or something else? Please elaborate (e.g., in the figure caption). Thank you for the reminder, the error bars represent the standard deviation. All figure captions were updated. 8.            Results and discussion, “disintegration rate in an industrial composting plant” subsection, bottommost paragraph. This paragraph needs revision. First, as mentioned above, it is likely that the mesh bag promoted mass transfer limitations on the biodegradation reaction. Second, I believe that the non-compliance of PLA to the EN 13432 standards is at least partially attributable to the high, 1 mm, thickness of the films and perhaps the high content of calcium silicate nanofiller may play a role as inhibitor of biodegradation. Are the composting conditions and medium a factor? I find that the inclusion of a positive control into the experimental design, such as a standard plastic material (e.g., a thinner PLA film) improves in the interpretation of the results, although I realize that the ISO standardized tests for disintegration do not directly call for it. Please consider revising the discussion in this paragraph. We tested the blends in the current form as they were prototypes of soft as well as rigid packaging respectively. We acknowledge the role of the blend composition and shape on the results. We appended a note to that paragraph and discussed the potential effects of the sample thickness in the Section “Feasibility of treating compostable plastic packaging in industrial composting plants” , paragraph 4. A comparison considering the filler content was added in the section “Disintegration rate in lab-scale industrial composting”, paragraph 3. 9.            Feasibility of treating compostable plastic packaging .. “ subsection, first paragraph, “do not meet the 90% disintegration requirement”. I find this paragraph requires revision, noting first that the PBS material does appear to meet the requirement under laboratory conditions, per Fig 2. The assessment given in this paragraph needs to take into account the high thickness of the PLA film and the mass transfer limitations imposed by the mesh bag for the data of Fig 3 (as discussed above) We understand and appreciate the comment. The section was revised to highlight the potential effects of sample thickness in paragraph 4. In addition, the wording was enhanced in order to make clear that the results do not represent an absolute negative evaluation but point out the many factors that needs to be additionally considered. A discussion of the potential effect of the mesh bags was also added to the section “Disintegration rate in an industrial composting plant”, paragraph 5 and reinterated in this section.

# Reviewer report: Reviewer response for version 1

**DOI:** 10.21956/openreseurope.16095.r29962

**Published:** 2022-10-10

**Authors:** Mehlika Karamanlioglu

**Affiliations:** 1 Faculty of Engineering and Architecture, Department of Biomedical Engineering, Istanbul Gelisim University, İstanbul, Turkey

This study compares degradation rate of compostable blends under lab conditions and in industrial composting plants. For this purpose, two different packaging blends, i.e., polylactic acid (PLA) based rigid packaging and polybutylene succinate (PBS) based soft packaging, were used. Lab-scale tests were carried out according to ISO 20200 and samples were recovered after 4, 8 and 12 weeks. On field test lasted for 3 weeks.  Both were under thermophilic conditions. Mass loss of samples was calculated to determine degradation.

1. In order to make an accurate comparison of degradation rates between lab conditions and on field conditions, sample recovery time should be the same. Samples were recovered earlier from the industrial composting site compared to lab scale tests. PLA quickly degrades under thermophilic conditions, above its glass transition temperature (T _g_), therefore one more week in an industrial composting site under thermophilic conditions would affect the results. It is mentioned that thermophilic phase does not exceed 3 weeks in industrial processes, therefore, samples could have been recovered during the 3 ^rd^ week of incubation under lab conditions as well so that an accurate comparison between lab conditions and on field conditions can be conducted. Otherwise, there is a duration factor under thermophilic conditions which cannot be omitted. Actually, for better comparison and for research purposes, on field samples should have been collected after 4, 8 and 12 weeks as well.
2. Different dimensions of each blend are used for each degradation environment. To make an accurate comparison of degradation rates between lab conditions and on field conditions, sample dimensions should be the same.
3. Apart from mass change, another degradation assessment method is highly recommended such as a Gel Permeation Chromatography (GPC). Cleaning the debris without compromising the sample is not easy which may lead to inaccurate results. For instance, molecular weights can be compared after 3 weeks of incubation in both conditions.
4. In methods, more detail about the plastics used in this study should also be given in this study as well (Table 1). The information would help to explain the degradation rate of the plastics. For instance, PLA properties such as its molecular weight and T _g_ greatly affect its degradation rate.
5. A brief comparison between the difference in degradation rate of PLA and PBS can be discussed.
6. Page 3: Please add the reference after these sentences: ‘Certified compostable starch-based shopping bags and PLA-based cutlery degraded by 48 wt% and 15 wt% respectively after a combination of a pilot-scale mesophilic dry anaerobic digestion phase (35 days), active composting phase (15 days) and compost maturation phase (40 days). A final concentration of compostable plastic in compost was found to be around 18 wt%.’
7. Page 6: Section 3.3 is mentioned but the section number cannot be found in the main text.
8. Page 8: ‘However, due to the very low disintegration rates observed, i.e. all samples were lower than 7%, it is unlikely that the disintegration rates will improve above the 90% disintegration rate required by ISO 20200 and EN 13432.’ The assumption drawn here is highly speculative since there is not enough data. No rate is determined, no interpolation is calculated and there are no mathematical models, etc. It is based on only one time sampling at the end of 3 ^rd^ week. Sampling should have been carried out each week  and for a longer period of time to make an estimation. 
9. Page 9: Grammar should be revised in the following sentence: ‘Further research on the effects of differing composting medium compositions, organic solids content, and particle size distributions are needed to understand the scenarios in which the disintegration rates will meet requirements’.
10. Page 10: In conclusion part, moisture content is another factor that affects degradation. Also, microorganisms and their diversity affect degradation in compost. They should be mentioned.
11. In conclusion part (Page 1), comparing 3 ^rd^ week in the field and 4 ^th^ week in the lab is not feasible. Why do the authors think the main reason of different degradation rate is the ‘composition of the composting substrate’. The evidence should be provided.
  In the same part, the last sentence should be more clearly presented.
12. In general, this research can be a great contribution to the literature and to the process of industrial composting of certain plastics. The research is clearly and accurately presented but some more fundamental and also recent papers should be cited to better explain the degradation of the plastics used in this study. Main factors that contribute to their degradation should be presented from the literature.
  In literature, PBS and even high molecular PLA degrades fast under thermophilic conditions in compost (Kale et al., 2007). However, in this study less than 5% of PLA blend was degraded in 3 weeks. Weren't samples in full contact with the compost due to mesh bags? Weren't they buried in the pile? The reasons of slower degradation rate in this study should be discussed more in detail and compared with literature. Is there any other study that contained samples in mesh bags, how long did it take for them to degrade?

A significant revision and some additional data mentioned above are necessary for this study.

Is the study design appropriate and does the work have academic merit?

Yes

Is the work clearly and accurately presented and does it cite the current literature?

Partly

If applicable, is the statistical analysis and its interpretation appropriate?

Not applicable

Are all the source data underlying the results available to ensure full reproducibility?

Yes

Are the conclusions drawn adequately supported by the results?

Partly

Are sufficient details of methods and analysis provided to allow replication by others?

Yes

Reviewer Expertise:

Biotechnology, biopolymers, environmental degradation of plastics.

I confirm that I have read this submission and believe that I have an appropriate level of expertise to confirm that it is of an acceptable scientific standard, however I have significant reservations, as outlined above.

## References

[1] : Comparison of the degradability of poly(lactide) packages in composting and ambient exposure conditions. *Packaging Technology and Science* .2007;20(1) : 10.1002/pts.742 49-70 10.1002/pts.742

# Response

**Published:** 2023-08-25

**Authors:** Zhi Kai Chong

**Affiliations:** Hamburg University of Technology, Germany

The authors would like to thank Mehlika Karamanlioglu for the detailed review and constructive comments. The table below summarises the authors’ comments and enhancements made to the new version in response to the review. Review Section Authors’ replies and comments (1) In order to make an accurate comparison of degradation rates between lab conditions and on field conditions, sample recovery time should be the same. Samples were recovered earlier from the industrial composting site compared to lab scale tests. PLA quickly degrades under thermophilic conditions, above its glass transition temperature (Tg), therefore one more week in an industrial composting site under thermophilic conditions would affect the results. It is mentioned that thermophilic phase does not exceed 3 weeks in industrial processes, therefore, samples could have been recovered during the 3rd week of incubation under lab conditions as well so that an accurate comparison between lab conditions and on field conditions can be conducted. Otherwise, there is a duration factor under thermophilic conditions which cannot be omitted. Actually, for better comparison and for research purposes, on field samples should have been collected after 4, 8 and 12 weeks as well.   (2) Different dimensions of each blend are used for each degradation environment. To make an accurate comparison of degradation rates between lab conditions and on field conditions, sample dimensions should be the same. The lab-scale test was done mainly to check the disintegration potential of the samples in a controlled environment. However, we completely understand and agree with the downside to the duration mismatch for comparison between the two types of tests. To facilitate a better comparison between the industrial-scale and lab-scale tests, an additional test in the lab based on ISO 20200 was conducted with a duration of 3 weeks to match the duration exposed in the industrial plant and added to the new version of the paper. Sample dimensions identical to those in the field test were used. The results and discussions were reevaluated based on the new data and described in the Section “Disintegration rate in an industrial composting plant”. It was observed that the lab-scale test according to ISO 20200 showed higher degradation and disintegration after 3 weeks. Conducting an experiment in the industrial plant for 4, 8 and 12 weeks was unfortunately not currently possible due to the high complexity of integrating the sample exposure method into the workflow of the plant. This is a potential point for future research. (3) Apart from mass change, another degradation assessment method is highly recommended such as a Gel Permeation Chromatography (GPC). Cleaning the debris without compromising the sample is not easy which may lead to inaccurate results. For instance, molecular weights can be compared after 3 weeks of incubation in both conditions. That is a really good point, however, a GPC test is not currently possible from our side. In our paper, the main measure of degradation is the mass loss with a cutoff threshold > 2 mm and we based our discussion on this measure. (4) In methods, more detail about the plastics used in this study should also be given in this study as well (Table 1). The information would help to explain the degradation rate of the plastics. For instance, PLA properties such as its molecular weight and Tg greatly affect its degradation rate. The average molecular weight was added for BPE-RP-PLA whilst the glass transition temperature was added for both BPE-RP-PLA and BPE-SP-PBS in Table 1. The average molecular weight for BPE-SP-PBS is not available. In addition, the effect of glass transition temperature on degradation was added to paragraph 7 of the Section “Introduction”.   The starting molecular weight of BPE-RP-PLA (237000 g/mol) was similar to a study (Kale et al., 2007) with 230000 g/mol, which reported complete disintegration within 30 days in a 65°C environment. However, the blend composition is different and the thickness was not mentioned in the study. Thus, making a direct comparison is not possible. In our study, the samples disintegrated significantly in the lab-scale (58 °C). On the other hand, degradation was observed with minor disintegration (mass loss) after the on-field test (64 °C). We explained possible reasons for this discrepancy in the Section “Differences in composting conditions between the lab-scale and on-field tests”. (5) A brief comparison between the difference in degradation rate of PLA and PBS can be discussed.   Thank you for the comment, a section discussing the contributing factors to the differing disintegration rate of BPE-RP-PLA and BPE-SP-PBS was added in Paragraph 3 of the Section “Disintegration rate in lab-scale industrial composting”. (6) Page 3: Please add the reference after these sentences: ‘Certified compostable starch-based shopping bags and PLA-based cutlery degraded by 48 wt% and 15 wt% respectively after a combination of a pilot-scale mesophilic dry anaerobic digestion phase (35 days), active composting phase (15 days) and compost maturation phase (40 days). A final concentration of compostable plastic in compost was found to be around 18 wt%.’ The sentence was rephrased mentioning the source, which was (Cucina et al., 2021). (7) Page 6: Section 3.3 is mentioned but the section number cannot be found in the main text. “Section 3.3” was corrected to “Differences in composting conditions between the lab-scale and on-field tests”. (8) Page 8: ‘However, due to the very low disintegration rates observed, i.e. all samples were lower than 7%, it is unlikely that the disintegration rates will improve above the 90% disintegration rate required by ISO 20200 and EN 13432.’ The assumption drawn here is highly speculative since there is not enough data. No rate is determined, no interpolation is calculated and there are no mathematical models, etc. It is based on only one time sampling at the end of 3rd week. Sampling should have been carried out each week  and for a longer period of time to make an estimation. The statement was removed. (9) Page 9: Grammar should be revised in the following sentence: ‘Further research on the effects of differing composting medium compositions, organic solids content, and particle size distributions are needed to understand the scenarios in which the disintegration rates will meet requirements’. The statement was rephrased to “However, research on the effects of differing substrate composition, particle size distribution and other parameters such as total volatile solids is lacking and important to understand the requirements for sufficient disintegration in actual plants as well as to understand the potential differences between plants.” (10) Page 10: In conclusion part, moisture content is another factor that affects degradation. Also, microorganisms and their diversity affect degradation in compost. They should be mentioned. Thank you for the comment, the factors were mentioned as suggested and added in the conclusion. (11) In conclusion part (Page 1), comparing 3rd week in the field and 4th week in the lab is not feasible. Why do the authors think the main reason of different degradation rate is the ‘composition of the composting substrate’. The evidence should be provided.   In the same part, the last sentence should be more clearly presented. The conclusion was revised and further elaborated addressing the comment with the new data from 3 weeks composting in the lab-scale: “Based on the data in this study, the abiotic conditions such temperature, moisture content and pH in the lab-scale was not notably better compared to on-field. The methods used also ensured that at least 1 kg of composting substrate was in direct contact with the plastic samples in all cases. This point towards the potential role of other composting parameters such as the process scale, substrate composition and particle size distribution in the rate of disintegration. These factors may vary for different plants and seasonally. In addition, the presence and diversity of microorganisms play a role.” (12a) In general, this research can be a great contribution to the literature and to the process of industrial composting of certain plastics. The research is clearly and accurately presented but some more fundamental and also recent papers should be cited to better explain the degradation of the plastics used in this study. Main factors that contribute to their degradation should be presented from the literature.   (12b) In literature, PBS and even high molecular PLA degrades fast under thermophilic conditions in compost (Kale et al., 2007). However, in this study less than 5% of PLA blend was degraded in 3 weeks. Weren't samples in full contact with the compost due to mesh bags? Weren't they buried in the pile? The reasons of slower degradation rate in this study should be discussed more in detail and compared with literature. Is there any other study that contained samples in mesh bags, how long did it take for them to degrade? Thank you for the comment. To enhance the new version, fundamental papers on degradation mechanics as well as more comparable composting studies were described and cited in the Section “Introduction”: paragraph 5 to 8. In addition, paragraphs 2, 3 and 4 of the Section “Differences in composting conditions between the lab-scale and on-field tests” were enhanced.   The authors think that this risk is minimal as the samples in this study were fully in contact with the organic waste as 1 kg of organic waste was enclosed together with the samples within the mesh bags. These bags were then placed in cages together with more organic waste. The cages were buried in the middle of the composting pile.   A summary of other studies was added to Paragraph 5 and 6 on the introduction. In addition, a comparison with other studies of industrial composting was added to paragraph 6 of the Section “Disintegration rate in an industrial composting plant” though it should be noted that a direct comparison of on-field composting studies is difficult due to the differing sample dimensions, blend composition, substrate composition and general composting methods.   References Cucina, M., Nisi, P. de, Trombino, L., Tambone, F., & Adani, F. (2021). Degradation of bioplastics in organic waste by mesophilic anaerobic digestion, composting and soil incubation. Waste Management (New York, N.Y.), 134, 67–77. https://doi.org/10.1016/j.wasman.2021.08.016 Kale, G., Auras, R., Singh, S. P., & Narayan, R. (2007). Biodegradability of polylactide bottles in real and simulated composting conditions. Polymer Testing, 26(8), 1049–1061. https://doi.org/10.1016/j.polymertesting.2007.07.006
